# Supplementary material for: Impact of COVID-19 restrictions on preschool children’s eating, activity and sleep behaviours: a qualitative study
Source: BMJ Open. 2021 Oct 17;11(10):e051497. doi: 10.1136/bmjopen-2021-051497 (PMC8523958; doi:10.1136/bmjopen-2021-051497)
Supplement: Supplementary data [file bmjopen-2021-051497supp001.pdf]

Coronavirus Preschool Study: a qualitative study of the lived experience of final year pre-school children in the UK during the coronavirus (COVID-19) disruption – Interview Topic Guide

Introduction

- Hello [name], it's [name] from the University of Bristol/Birmingham/Glasgow speaking, how are you doing? Are you still ok to chat to me today? It will take up to an hour.
- Thank you so much for sparing your time to speak to me today.
- Before starting the interview, I'm going to talk a bit about the study. I'm then going to take your consent before we start the interview.
- Firstly, just to give a bit of background of the study, we want to find out about how pre-school children are doing during the coronavirus disruption, and what effects, if any, it is having on their mood and their physical activity and eating behaviours. Throughout the interview I will refer to coronavirus which some people call COVID-19.
- When answering the questions, please try to focus on your final year pre-school child rather than any other children in the household.
- We are interested in the whole period since mid-March, i.e. lockdown, easing of restrictions and now.
- Feel free to ask questions at any time.
- There are no wrong or right answers and you do not have to answer all of the questions if you do not want to.
- Anything that you tell me is confidential and will not be linked to you. The only reason for me needing to break confidentiality would be if you say something where I am concerned about harm to you or someone else.
- You can stop at any time and if you decide in the next two weeks that you do not want me to include what you said, just let me know.
- I have already sent you a copy of the consent form along with the participant information sheet. When I start the voice recorder, I'm just going to read out each statement on the consent form one by one and if you could please confirm that you're happy with each statement. This is something we have to do as part of the University ethics regulations.
- Do you have any questions before I start the recording? If it's ok, I will start recording now.

START RECORDING

- State interviewer name, participant name, participant ID number and date/time of interview.
- Read out each statement on the consent form. Ask participant to say 'Yes' after each statement if they are happy with the statement.
- Explain to the participant that you are now going to stop the audio-recording, and re-start recording for the main part of the interview.

STOP RECORDING

START RECORDING

State interviewer name, participant ID and date/time of interview (do NOT state participant name).

Clarke J, *et al. BMJ Open* 2022; 11:e051497. doi: 10.1136/bmjopen-2021-051497

Background

Tell me about your household	• Name/age/gender of child focusing on for this interview<br>• Relationship of interviewee to child<br>• Age/gender of any other children<br>• Do you have a garden?
Is anyone in the house working ?	• Any keyworkers ?<br>• Anyone working from home; furloughed?
Has anyone in the household had coronavirus ?	• Tested or untested

Childcare

Remind participant that the focus of the interview is on the final year preschool child (not on other children in the family).

**Before** the coronavirus disruption, what were you doing for childcare?	• Setting (e.g. nursery, childminder, family members)<br>• How many days/hours per week of childcare?
**During** the lockdown when nurseries were shut except to key workers, did your child attend any childcare?	• If so, why? As much as before, or less?
How have you been managing childcare with the coronavirus disruption?	• Who is/has been looking after the children?<br>• If your nursery has **re-opened**, is your child currently attending any childcare? Is this as more or less than before?<br>• If your child has not returned to childcare, can you tell me why?<br>• If you child has returned to childcare, what changes have been made in childcare because of coronavirus? (e.g. attending part-time, parents not allowed in, smaller groups, changes to food provision)

Your child's mood and how they have been feeling

note to interviewer – we are interested in the whole period since mid-March, i.e. lockdown, easing of restrictions and now

How has your child been feeling about the coronavirus restrictions?	• What effects (if any) do you think the restrictions have had on your child's well-being?<br>• Have the restrictions had any effect on your child's mood and feelings? (positive or negative)<br>• Have you noticed any changes at all in your child's behaviour? (positive or negative)<br>• What is the impact on the child of any reduced social contact with other children, friends and family?

Clarke J, *et al. BMJ Open* 2022; 11:e051497. doi: 10.1136/bmjopen-2021-051497

Physical activity

We're interesting in how the lockdown and the social distancing restrictions that are happening now might have affected young children's activities they do when they're moving around or sat still. By moving around, I mean anything from: Pottering (e.g. Lego, helping around the house, small-world play, dressing up); On the Go: (e.g. playing in the garden, dancing, hide and seek, playing on the furniture, rough and tumble); and Huff and Puff: (e.g. running games, trampolining, scooting, dancing).

note to interviewer – we are interested in the whole period since mid-March, i.e. lockdown, easing of restrictions and now

What activities has your child been doing during the coronavirus disruption?	• Has the coronavirus disruption had any effect on how active your child is?<br>• What has changed? What has stopped?
During the time since lock-down started in March has your child been going out of your home and garden (if they have one)?	• If not, why not?<br>• Has this changed as lockdown has been eased?
Do you think your child is currently more or less active than before the disruption?	• Why?<br>• How do you think any changes will affect how active your child is in the future?<br>• Will you re-start any activities that you can no longer do?<br>• Will you carry on doing any new activities?
What sitting activities has your child been doing during the coronavirus disruption? (e.g. screen time, educational activities, TV, games, social interaction)	• Has there been any change in the amount of time the child spends sitting?<br>• Have there been any changes in screen time rules or supervision of screen time in your house during the disruption?<br>• How do you think any changes will affect future screen use?
Do you think the changes since March due to coronavirus have affected your child's sleep?	• In what way?

School

How are you feeling about your child starting school in September?	• How is your child feeling?<br>• Any thoughts about whether child will start in September, or whether you may delay starting school?
Are there any things you would have been doing to help prepare the child for starting school that you aren't able to do because of the disruption?	• E.g. visits to new school, end of preschool special events<br>• What could help with the transition during this time of uncertainty?

Nutrition

note to interviewer – we are interested in the whole period since mid-March, i.e. lockdown, easing of restrictions and now

Have there been any changes in what your child has been eating because of the coronavirus disruption?	• E.g. changes in quantity of food, types of food?<br>• Any changes in how you get your food, what food you can get, how much you spend?<br>• Changes in snacking/treats?<br>• Any changes to food preparation at home? E.g. more/fewer take away meals, cooking with child, cooking from scratch?<br>• Changes in fruit and vegetable consumption?
Overall, do you think that your child is eating more or less healthily than before the disruption?	• Why?<br>• Thinking about the future, do you think that any changes to your child's eating will continue?

Demographic questions

- We would like to ask you some questions about you and your children. If you do not wish to answer, please just say so.
- If you don't mind telling me, what is your age?
- How would you describe your ethnicity?
- Out of the following options, what is your current employment status?
    - Student
    - Stay at home parent/caregiver
    - Full-time
    - Part-time
    - Unemployed
    - Furloughed
- What is your highest level of education? Options:
    - Up to GCSEs/GCEs/O levels or similar
    - A levels/NVQs/GNVQs
    - First degree/diploma/HNC/HND
    - Higher degree (e.g. MSc, PhD)
- Finally, how did you find out about this research study i.e. where did you see the study advert?

End

- That's all the questions I have for you today. Do you have any questions for me?
- I will stop recording the interview now.

STOP RECORDING

Thank the participant and explain that a voucher will be emailed to them within 7 days.

Clarke J, *et al. BMJ Open* 2022; 11:e051497. doi: 10.1136/bmjopen-2021-051497
