# Supplementary material for: Impact of COVID-19 restrictions on preschool children’s eating, activity and sleep behaviours: a qualitative study
Source: BMJ Open. 2021 Oct 17;11(10):e051497. doi: 10.1136/bmjopen-2021-051497 (PMC8523958; doi:10.1136/bmjopen-2021-051497)
Supplement: Author's manuscript [file bmjopen-2021-051497.draft_revisions.pdf]

BMJ Open is committed to open peer review. As part of this commitment we make the peer review history of every article we publish publicly available.

When an article is published we post the peer reviewers' comments and the authors' responses online. We also post the versions of the paper that were used during peer review. These are the versions that the peer review comments apply to.

The versions of the paper that follow are the versions that were submitted during the peer review process. They are not the versions of record or the final published versions. They should not be cited or distributed as the published version of this manuscript.

BMJ Open is an open access journal and the full, final, typeset and author-corrected version of record of the manuscript is available on our site with no access controls, subscription charges or pay-per-view fees (http://bmjopen.bmj.com).

If you have any questions on BMJ Open's open peer review process please email info.bmjopen@bmj.com

BMJ Open

Impact of COVID-19 restrictions on pre-school children's eating, activity and sleep behaviours: a qualitative study

Journal:	*BMJ Open*
Manuscript ID	bmjopen-2021-051497
Article Type:	Original research
Date Submitted by the Author:	23-Mar-2021

Complete List of Authors:	Clarke, Joanne; University of Birmingham, Institute of Applied Health Research<br>Kipping, Ruth; University of Bristol, Centre for Public Health, Population Health Sciences, Bristol Medical School<br>Chambers, Stephanie; University of Glasgow, School of Social and Political Sciences and MRC/CSO Social and Public Health Sciences Unit, Institute of Health and Wellbeing<br>Willis, Kate; University of Bristol, Centre for Public Health, Population Health Sciences, Bristol Medical School<br>Taylor, Hilary; University of Bristol, Centre for Public Health, Population Health Sciences, Bristol Medical School<br>Brophy, Rachel; University of Bristol, Centre for Public Health, Population Health Sciences, Bristol Medical School<br>Hannam, Kimberly; University of Bristol, Centre for Public Health, Population Health Sciences, Bristol Medical School<br>Simpson, Sharon; University of Glasgow, School of Social and Political Sciences and MRC/CSO Social and Public Health Sciences Unit, Institute of Health and Wellbeing<br>Langford, Rebecca; University of Bristol, Centre for Public Health, Population Health Sciences, Bristol Medical School

Keywords:	COVID-19, Community child health < PAEDIATRICS, NUTRITION & DIETETICS, QUALITATIVE RESEARCH

SCHOLARONE™
Manuscripts

I, the Submitting Author has the right to grant and does grant on behalf of all authors of the Work (as defined in the below author licence), an exclusive licence and/or a non-exclusive licence for contributions from authors who are: i) UK Crown employees; ii) where BMJ has agreed a CC-BY licence shall apply, and/or iii) in accordance with the terms applicable for US Federal Government officers or employees acting as part of their official duties; on a worldwide, perpetual, irrevocable, royalty-free basis to BMJ Publishing Group Ltd ("BMJ") its licensees and where the relevant Journal is co-owned by BMJ to the co-owners of the Journal, to publish the Work in this journal and any other BMJ products and to exploit all rights, as set out in our licence.

The Submitting Author accepts and understands that any supply made under these terms is made by BMJ to the Submitting Author unless you are acting as an employee on behalf of your employer or a postgraduate student of an affiliated institution which is paying any applicable article publishing charge ("APC") for Open Access articles. Where the Submitting Author wishes to make the Work available on an Open Access basis (and intends to pay the relevant APC), the terms of reuse of such Open Access shall be governed by a Creative Commons licence – details of these licences and which Creative Commons licence will apply to this Work are set out in our licence referred to above.

Other than as permitted in any relevant BMJ Author's Self Archiving Policies, I confirm this Work has not been accepted for publication elsewhere, is not being considered for publication elsewhere and does not duplicate material already published. I confirm all authors consent to publication of this Work and authorise the granting of this licence.

For peer review only

Impact of COVID-19 restrictions on pre-school children's eating, activity and sleep behaviours: a qualitative study

Authors:

Clarke JL[1], Kipping R[2], Chambers S[3], Willis K[2], Taylor H[2], Brophy R[2], Hannam K[2], Simpson SA[4] Langford R[2]*

[1] Institute of Applied Health Research, University of Birmingham

[2] Centre for Public Health, Population Health Sciences, Bristol Medical School, University of Bristol

[3] School of Social and Political Sciences and MRC/CSO Social and Public Health Sciences Unit, Institute of Health and Wellbeing, University of Glasgow

[4] MRC/CSO Social and Public Health Sciences Unit, Institute of Health and Wellbeing, University of Glasgow

*corresponding author: beki.langford@bristol.ac.uk, Canynge Hall, 39 Whatley Road, Bristol, BS8 2PL, UK.

For peer review only

Abstract

Objectives: In spring 2020, the COVID-19 lockdown placed unprecedented restrictions on the behaviour and movements of the UK population. Citizens were ordered to 'stay at home', only allowed to leave their houses to buy essential supplies, attend medical appointments or exercise once a day. We explored how lockdown and its subsequent easing changed young children's everyday activities, eating and sleep habits to gain insight into the impact for health and wellbeing.

Design: In-depth qualitative interviews; data analysed using thematic analysis.

Setting: South West and West Midlands of England.

Participants: Twenty parents (16 mothers; 4 fathers) of pre-school age children (3-5 years) due to start school in September 2020. Half the sample were from Black, Asian or Minority Ethnic backgrounds and half lived in the most deprived quintile.

Results: Children's activity, screen time, eating, and sleep routines had been disrupted. Parents reported children ate more snacks, but families also spent more time preparing meals and eating together. Most parents reported a reduction in their children's physical activity and an increase in screen time, which some linked to difficulties in getting their child to sleep. Parents sometimes expressed guilt about changes in activity, screen time and snacking over lockdown. Most felt these changes would be temporary, though others worried about re-establishing healthy routines.

Conclusions: Lockdown negatively impacted on pre-school children's eating, activity and sleep routines. While some positive changes were reported, many participants reported of lack of routines, habits and boundaries which may have been detrimental for child health and development. Guidance and support for families during COVID-19 restrictions could be valuable to help maintain healthy activity, eating, screen-time and sleeping routines to protect child health and ensure unhealthy habits are not adopted.

Strengths and Limitations of this study

- Few studies have explored the impact of COVID-19 restrictions on young children or health behaviours like physical activity or diet.
- Our in-depth interviews provide novel insights into the impact of lockdown restrictions on pre-school children’s physical activity, sedentary behaviour, food intake and sleep.
- A strength of the study was the ethnic and socioeconomic diversity of the sample with half of participants from the lowest deprivation quintile and half from Black, Asian or Minority ethnicities.

For peer review only

- Interviews were conducted parents, rather than preschool children themselves, for pragmatic reasons.

Key words

COVID-19; coronavirus; pre-school; child; paediatric; physical activity; sleep; qualitative; diet; screen time

For peer review only

Background

The emergence of COVID-19, and subsequent efforts to restrict its spread, led to unprecedented economic and social disruption[1]. Many countries imposed restrictions on citizens' behaviours and movements to limit the spread of the virus, including social distancing and local or national 'lockdowns'.

The UK Government imposed its first 'lockdown' in March 2020. Citizens were required to 'stay at home' only leaving to buy essential supplies, attend medical appointments or to exercise. Schools and nurseries were closed (except to vulnerable or 'keyworker' children), as were leisure facilities, pubs, restaurants, theatres and non-essential shops. Non-essential workplaces were closed, and people could not meet anyone beyond their immediate household. Unlike more stringent lockdowns elsewhere, UK citizens were permitted to exercise outside once a day[2]. Restrictions lasted over 2 months until late May 2020. Pre-schools reopened to all children in June 2020, alongside schools who re-opened to certain year groups at this point; however, occupancy remained low with attendance half that recorded for the previous year (37% vs.77%) [3].

Despite being least affected by the virus[4] children have experienced enormous disruption which may have impacted their health behaviours. Children under five should have 180 minutes of physical activity a day [5]. Children aged 3-4 should have 11-13 hours sleep in a 24-hour period [6]. Lockdown reduced children's opportunities for physical activity, while increasing the likelihood of sedentary behaviours [7] and had the potential to disrupt sleep patterns [8]. The spring lockdown also changed people's food shopping and eating habits. Consumers moved to less frequent shopping trips, but spent more on food than pre-lockdown, potentially changing the home food environment [9].

Several surveys have sought to capture the impact of COVID-19 on children and young people's health and well-being. Most have focused on emotional wellbeing and have often targeted older children[10]. Less attention has focused on younger (pre-school) children or explored the impact on physical activity, sedentary behaviour and diet. An exception is the Co-SPYCE study [11] which surveyed

For peer review only

caregivers of 2-4 year olds. It found 26% of children had 3+ hours of screen time a day, and only 22% were active for 180 minutes/day. Public Health Scotland developed the COVID-19 Early Years Resilience and Impact Survey (CEYRIS) [12] for 2-7 year olds in Scotland. From 11,228 participants, eating behaviour was identified as worse by 32% of parents. There were mixed responses about physical activity, with 24% reporting children doing more during lockdown and 47% reported less.

We explored the impact of lockdown, and its easing, on pre-school children through qualitative interviews with parents focusing on: physical activity, sedentary behaviour, food intake and sleep. Establishing healthy behaviours in the early years is important in maintaining a healthy weight [13]. Nearly one in four UK children starting primary school are overweight or obese, with rates increasing with deprivation[14]. Many children saw time in childcare reduced or stop altogether [15]. Pre-school children require greater parental supervision which may mean managing children's activities and behaviours in lockdown was more challenging for these families, particularly if parents were working or there were additional family stressors [16].

Methods

Population, sampling and recruitment

Criteria for inclusion were: (1) parent/carer of a child aged 3-5 years in their final year at pre-school; (2) child usually attends pre-school at least once per week; (3) child due to start school in September. Non-English speakers were excluded for pragmatic reasons.

Parents were recruited via two methods. First, we asked nurseries participating in another research study [17] to disseminate study information to parents via email or social media. This included nurseries from Swindon and Somerset (South West England) and Sandwell (West Midlands). Second, we posted study information on local Facebook groups within these areas and Birmingham (West Midlands) to increase recruitment of Black, Asian and Minority Ethnic (BAME) participants. Interested parents (n=85) completed a form to check eligibility and collect postcode (deprivation) and ethnicity data for sampling

For peer review only

purposes. To provide diversity, sampling was weighted towards parents in the most deprived areas and those from BAME populations. Twelve parents were recruited via nurseries and eight via Facebook.

Sampled parents were emailed the participant information sheet (PIS) and consent form. Interviews were arranged with participants at least 24 hours after sending study documents. All sampled parents agreed to participate, bar one who we were subsequently unable to contact.

Patient and public involvement

Nursery staff provided advice on the best ways to recruit parents for this study.

Data collection

Semi-structured interviews were conducted by experienced qualitative researchers (JC, KW or SC) in July/August 2020 by telephone (n=18) or video (n=2). Before interview, the researcher explained the study aims, answered questions and gained consent. A topic guide (Table 1) was used to guide discussions with parents encouraged to talk openly about their experiences. Interviews were audio-recorded, transcribed verbatim and anonymised. Following each interview, notes were made of key points raised. A £30 shopping voucher was sent to participants following interview.

Data analysis

Data were analysed thematically [18] using NVivo (version 12) to aid data management and analysis. Transcripts were read by JC/RB/HT to gain familiarity with the data. Three transcripts were independently coded by these researchers to generate an initial list of codes. Codes were both deductive (generated from topic guide and research questions) and inductive (generated from interview data). Differences in coding were resolved through discussion to produce an agreed coding framework. Subsequent transcripts were single-coded using this framework with further discussion to clarify or expand the framework as needed.

For peer review only

Ethical approval

Ethical approval was granted by University of Bristol Faculty of Health Sciences Research Ethics Committee (ref: 106002).

Results

Participants

Sixteen mothers and four fathers participated in the study (Table 2). Average age of parents was 34 years (range 21-45 years). Half the sample were White British. Thirteen participants were educated to degree level. Half resided in the most deprived Index of Multiple Deprivation (IMD) quintile. Five participants were single parents. Thirteen reported at least one parent was not working during the lockdown period; three reported that no household member was currently employed. Two participants had no access to a garden.

Food and eating

Increased snacking

Most parents reported their child's snacking had increased over lockdown, which they often linked to stay-at-home restrictions. Children were bored and consequently snacked more: "*When there's nothing to occupy them, they've got to do something*" (Mother_1). Some felt loss of routine had disrupted rules around snacking: *"if he's at pre-school he can't snack. He can only eat at the specific snack time…Here, he just thinks it's on tap."* (Mother_2). Some found dealing with demands for snacks challenging, with some parents permitting more "unhealthy" items than normal: *"It's all we hear. She's constantly hungry… She's definitely eating more bags of crisps than she normally would"* (Mother_3).

Some parents created make-shift rules around food, balancing child demands with wanting to provide "healthy" snacks. Mother_4's children were allowed crisps because they went *"on and on"* but then were told *"to move on to the fruit bowl."* Another parent made healthy snacks more accessible: *"I make sure there are bits and bobs cut up in the fridge… If she's going to help herself to something, it's the*

For peer review only - http://bmjopen.bmj.com/site/about/guidelines.xhtml

stuff on the bottom she can reach" (Mother_5). However, some parents gave additional 'treats' to their children to compensate for the pandemic's impact: *"We were feeling bad they'd missed out on so much, we were buying treats to make them feel happy."* (Mother_6)

Food preparation and mealtimes

Some parents reported an increase in problematic mealtime behaviours, such as children not wanting to eat or taking a long time to eat. Often this was linked to increased snacking. However, several reported positive effects on mealtime routines. With parents working from home, families could eat together more often. Similarly, parents enjoyed having time to cook and involve their child. This helped occupy their child and taught them new skills: "*When we couldn't go to McDonald's we made homemade chicken nuggets… that's been positive, they've enjoyed cooking"* (Mother_6).

Lockdown had other impacts on families' food behaviours. With restaurants shut, people no longer ate out. Some participants also reported consuming less take-aways. For one, this was related to concerns about the virus: "*I didn't touch any takeaways. I didn't feel comfortable." (*Mother_4). In contrast, one parent reported eating more take-aways because "we *all need something to look forward to"* (Mother_7).

Food costs

Several participants reported spending more on food during lockdown. Some related this to the whole family being at home, as well as increased snacking, while others felt prices increased. One family no longer had access to free meals at their child's nursery. Another participant who was shielding had moved to on-line deliveries, a service unavailable at their normal cheaper supermarkets: "*We'd normally just go to Aldi or Lidl… now we've been trying to do the online stuff, it's just added to our bill*" (Mother_9). However, an unexpected benefit of this was healthier snacking: "*it's a money thing, we've not been able to buy all the junk food*" (Mother_9).

For peer review only

Physical activity

Most parents felt their child was less active during lockdown. For some this was substantial - *"we* [were] *nowhere near as active as we used to be"* (Mother_4) – while a minority felt it had little impact. Several related this reduction to childcare closures which removed usual opportunities for activity: *"I put it down to routine and being less active… At nursery, you're on the go from the time you get there to the time you go home"* (Mother_10). Opportunities for active transport to and from childcare were also lost: *"[She's] definitely less active because we walk to [pre-]school and that was 10 minutes there and 10 minutes back… She's not having that exercise*" (Mother_11). Normal activities (like swimming, soft play) were also closed and opportunities to socialise with others restricted.

Parents noted several factors that helped children stay active. Having a sibling was important, providing someone to play with. Equally, access to play equipment helped facilitate activity: *"They've got a trampoline, a bike each, a scooter each"* (Mother_4). Access to outside space was also important. Only two parents had no access to outdoor space, but both described this as challenging during lockdown. As Mother_8 explained, "*We couldn't leave and there was nothing really you could do. And living in a block of flats, you can't be too noisy because you've got neighbours everywhere*" (Mother_8). In contrast, Mother_1 noted how having a safe space to cycle outside her house helped keep her son active: "*My front* [garden] *is really nice for children to play and ride bikes."*

Good weather was also seen as an important facilitator of activity: "*We had the pool out and [he] was using it quite a lot … We were pretty much burning energy off most of the time, especially with the nice weather we had*" (Mother_12). Some parents discussed how they made use of the local environment during lockdown: *"We've done a lot of exploring around the local area… we've found some places we didn't know were there"* (Mother_6).

Parents often put effort into keeping their child physically active: *"I've tried really hard to make sure he's been as active as possible"* (Mother_7). This was easier for families where one parent was not

working: *"He has to have a parent with him. 'Look at me! Look at my star jumps!'… That was the difficult bit, because there is only so much of that you can do, especially when you've got work*." (Father_1)

Sedentary behaviours

Parents reported their child engaged in a range of sedentary activities during lockdown (like reading, drawing) but screen time was mentioned most frequently with almost all parents reporting substantial increases. For many families, screen time filled the void left by being unable to go out or socialise. Mother_13 explained they watched more television because "*we were stuck in the house… we weren't having that release of getting out",* while Mother_1 commented "[My son] *can't go to friends, he can't meet family… so the screen had become his friend".*

Many felt screen time had been useful during this difficult period, with one parent referring to it as a *"lifesaver"* (Father_1). For those trying to work from home, it was often the only way they could manage: *"If I've got a two-hour Zoom meeting and I don't want him bursting in every five seconds I'd give him his iPad and he'll watch a film*" (Mother_6). Even for those not working, managing everyday chores was a challenge: *"You've still got housework to do, washing to do, meals to prepare… You can't always be there 24/7 to keep her occupied"* (Mother_3).

Screen time was also used to provide much needed respite from the intensive parenting effort lockdown enforced. Father_2 described screen time as providing "*a break"* while Mother_11 used it to avoid constant sibling squabbles: *"They're just following you around … fighting and hitting each other… just sit down quietly and watch a movie."* However, parents' language suggested many felt uncomfortable about increased screen time. Mother_6 admitted *"It's bad, I don't even like saying it out loud, but [screen time] was almost like a pacifier, really*", while Mother_3 admitted "*It's going to sound horrible, but it's just sometimes easier".*

Parents often distinguished between 'good' and 'bad' screen time. Educational or interactive screen time was better than passive television watching. Mother_11 would ask herself "*Is it educational or is it*

For peer review only

just rubbish they're watching?", while Mother_3 explained "*I don't want her to mindlessly sit there watching a screen. At least if she's playing [educational] games, it's making her think.*"

Concerns over screen time were rarely linked to lack of movement or activity. Rather Father_3 referred to *"the effect of the brain becoming lazy."* Mother_5 suggested too much screen time interfered with normal childhood and fostered an inability to occupy oneself: "*They're not being children. I suppose you think about your own childhood, don't you? All the bits and bobs I used to do as a kid. When I've said no screen time, they just wander around like aimless sheep.*"

Some parents created new rules to manage increased screen time. This included restrictions on the amount, times of day or content. Mother_14 explained, "*We have had to impose rules… we've just had to structure it a bit more, because she would gladly sit in front of the telly or iPad all day.*" Other families took a more reactive approach: "*We're not a routine sort of family anyway, so if they've had an hour or so, then I'll be like, 'Right, that's it now, everybody out.'*" (Mother_5)

Sleep

Almost half of parents reported negative changes to their child's sleep. Many reported difficulties in getting their child to sleep, with some staying up very late. Parents often related this either to lack of activity – "*it's because she's not been as active and been out*" (Mother_8) – or increased screen time – "*a direct correlation of being a bit more bored, spending more time on a screen*" (Mother_3). Loss of routine was again a source of difficulty for some families. As Mother_4 commented: "*Before it was a very strict* [bedtime] *routine ... now it is kind of as and when*". A minority of parents wondered if anxiety played a part in their child's sleep difficulties: "*I'm just wondering whether* [his frequent waking] *was a part of maybe anxiety or worried about everything that's going on.*" (Mother_12)

Longer term impacts

Generally, parents thought children's increase in snacking during lockdown was "*just a temporary thing*" (Mother_2). As Mother_5 acknowledged: "*when she goes to school she's not going to have that*

For peer review only

opportunity to (a) steal food or (b) she won't be home to be eating masses during the day". However, one parent whose child had already returned to nursery acknowledged getting back into a routine was difficult: *"Trying to get him to eat properly once we started to go back into a routine was really hard"* (Mother_10).

Several parents wished to sustain positive changes established during lockdown, such as home-cooked meals and involving children in food preparation. However, these changes required time and would need a *"conscious effort to keep doing"* (Mother_14). Many talked positively about returning to previous activities (e.g. swimming, gymnastics) though several admitted feeling *"wary"* (Father_4) about how safe these were, especially those involving close contact.

Some parents were concerned about the impact of lockdown on their child's skills and confidence. Mother_14 felt her daughter had lost her swimming skills and noted a general loss of confidence: "*She's a lot more cautious on her bike because for a while they just didn't leave the house… she's had a step backwards with that.*" Another was concerned about lost stamina and wondered if her daughter would manage the mile-long walk to school: "*I am actually a little bit worried about how she's going to fare… she's not been used to doing that walk*" (Mother_5).

Most parents were not overly concerned about increased screen time, assuming it would naturally decrease with a return to routine. However, others wondered how an increased reliance on screens during lockdown might change their child's behaviours going forwards. Mother_6 described her son pre-lockdown as a *"pocket rocket*" who never sat still. Now, however, "*rather than going outside and running around the garden, as soon as he wakes up [he asks] 'Can I have the iPad?'…He would quite happily sit all morning.*" Similarly, Mother_3 explained her daughter would now *"rather have something on the telly than go out".* Allowing her daughter to watch television in her mother's bedroom during lockdown, Mother_3 acknowledged the difficulty in re-establishing pre-lockdown boundaries: "*She's asking Santa for a television for her bedroom, which I'm hoping she forgets.*"

For peer review only

Discussion

During the first COVID-19 lockdown parents reported increased snacking, screen time and sleep issues, and decreased physical activity in their pre-school children. There were also positive family behaviour changes, such as more eating together and use of the local environment for physical activity. Some parents expressed concerns about the future impact of negative behaviour changes, but most believed these were a temporary consequence of a lack of routine.

This study provides in-depth reports of family life with a pre-school child during the pandemic, and complements findings of parents of 2-4 year olds from the Co-SPYCE survey [11] which revealed children's screen time as a key parental stressor during lockdown. Parental use of children's screen time to 'get things done' was a key finding of a recent qualitative systematic review [19]. In our study, with the absence of childcare, and corresponding increase in parental juggling of work and household chores, it is clear how this 'need' for downtime increased for many.

Most parents felt their child was less active over lockdown echoing findings from other studies. Only 22% of 2-4 year olds in the Co-SPYCE study were getting 180 minutes of activity per day [20] and almost half (47%) of Scottish parents reported their children (2-7yrs) did less physical activity during lockdown [12]. A Canadian survey of parents of 5-17 year olds found children were less active and engaged in more screen time during the initial COVID-19 outbreak compared with before restrictions. Parent encouragement, participation and support of physical activity were found to be important correlates of children's physical activity [21]. Zecevic et al [22] showed that, in circumstances not associated with lockdown, young children who receive parental support for physical activity were more likely to be highly active than inactive. In our study some parents made considerable efforts to keep their child active. Yet despite these efforts, barriers specific to lockdown were reported which reduced activity, mainly relating to the closure of childcare and playgrounds, and reduced contact with friends and family.

Our study identified disruptions to normal eating habits and routines including changes in snacking behaviour, frequency and volume of eating and who children were eating with. While some families

For peer review only

speculated these changes would soon return to normal, it may be difficult to re-establish boundaries and break new habits [23,24]. One parent discussed how free meals for her child at nursery had ceased. While our study did not identify acute food poverty, it is likely that pre-school children will have experienced food poverty where free school meals were no longer available and where the pandemic impacted family finances [25].

Rundle et al (2020) suggested the closure of schools during this period may exacerbate childhood obesity levels[26]. While few parents mentioned weight, our findings suggest there is potential for obesity prevalence in pre-school children to have increased due to increased snacking and screen time, and decreased physical activity, particularly if changes during lockdown have persisted. The annual National Child Measurement Programme was stopped in March 2020 but restarted in January 2021. This will enable assessment of the impact on children who started school in September 2020 [27].

The negative impacts of COVID-19 are expected to be greatest for children living in poverty[28,29]. We sought to explore differences in the impact of lockdown across the socioeconomic spectrum. Half our participants lived in the most deprived IMD quintile, giving insight into those living in the poorest areas. We noted some differences within our sample. Two families without access to gardens reflected on the difficulty in keeping children active. Families where parents were not working found it easier to engage children in physical activity. Beyond this, however, we did not note any obvious differences between those living in more or less deprived neighbourhoods. The impact of lockdown on children's behaviours was felt similarly across the socioeconomic spectrum. The lack of difference could be due to sample characteristics; most families still had at least one parent working and access to a garden. Interviews took place early in the COVID-19 crisis, so we may not yet be seeing the full effect, for example with the protective effect of the furlough scheme still in place.

This study raises questions about how parents can be supported during future lockdowns or local restrictions. Subsequent UK lockdowns in late 2020 and early 2021 presented new and additional challenges: shorter, colder days, weariness of the restrictions, and increased financial pressures with high

For peer review only

rates of unemployment and redundancy[30]. In addition, positive cases in childcare or school settings can result in children being required to isolate for 14 days without being allowed out. The importance of supporting families to maintain or increase activity for young children within the home and in accessible outdoor environments becomes more prominent. Families need support to establish healthy revised routines and manageable healthy rules for snacks and screen time during periods of restriction. This must be presented in a context of supporting parents and not adding guilt or burden to parents during a period of stress. A recent Ipsos Mori report, which included an online survey of 1,000 parents of 0-5s, found 70% of parents felt judged by others and almost half felt this negatively impacted on their mental health [31]. There is a role here for all sectors of health, social care and education including health visitors, children's centres, early years providers, GPs, social services, local authority public health teams and Public Health England. Further, there is a role for research with pre-schoolers and 2020 school starters to be expanded [32] to understand the impact on their physical and social development, and particularly their risk of obesity, as they start school.

Strengths and limitations

In-depth interviews enabled us to gain novel insights into the experiences of parents and pre-school children during lockdown and restrictions, though we acknowledge the limitation of investigating pre-school children's experiences by asking their parents, rather than involving pre-school children directly. A strength of the study was the ethnic and socioeconomic diversity of the sample from two regions of England, although this was a relatively educated sample. Parents volunteering for interview may have had more interest in the topic or had different experiences to those who did not volunteer. Rigour was achieved by detailed data analysis and analytical decisions being shared with all team members to achieve credibility.

Conclusions

While some positive changes were reported, there were wide-spread examples of lack of routines, habits and boundaries which, at least in the short-term, were likely to have been detrimental for child health and

For peer review only

development. Guidance and support for parents and families on how to maintain healthy routines and compensate for future COVID-19 restrictions could be valuable to protect child health and ensure that unhealthy habits are not adopted.

For peer review only

Licence statement

I, the Submitting Author has the right to grant and does grant on behalf of all authors of the Work (as defined in the below author licence), an exclusive licence and/or a non-exclusive licence for contributions from authors who are: i) UK Crown employees; ii) where BMJ has agreed a CC-BY licence shall apply, and/or iii) in accordance with the terms applicable for US Federal Government officers or employees acting as part of their official duties; on a worldwide, perpetual, irrevocable, royalty-free basis to BMJ Publishing Group Ltd ("BMJ") its licensees and where the relevant Journal is co-owned by BMJ to the co-owners of the Journal, to publish the Work in BMJ Open and any other BMJ products and to exploit all rights, as set out in our licence.

Abbreviations

BAME	Black, Asian and Minority Ethnic
CO-SPYCE	Covid-19: Supporting Parents and Young Children during Epidemics (study)
COVID-19	Coronavirus Disease (also known as SARS-CoV-2)
GPs	General Practitioners
IMD	Index of Multiple Deprivation
UK	United Kingdom

Ethics approval and consent to participate

Ethical approval for the study was granted by University of Bristol Faculty of Health Sciences Research Ethics Committee. Participant consent was recorded on a separate audio file before the interview commenced.

For peer review only

Consent for publication

Consent to include anonymised information in publications was given by all participants.

Availability of data and materials

Anonymised study data will be made available via a University of Bristol repository.

Competing interests

The authors declare that they have no competing interests.

Funding

This work was funded by the NIHR School for Public Health Research (SPHR-PROG-CYP-WP3) and NIHR funding for the NAP SACC UK trial (2019-3426).

Authors' contributions

The study was conceived by JC, RK, SC, KW, HT, RB, KH, SS and RL. JC led the study with oversight from RL and RK. Interviews were conducted by JC, SC and KW. Coding of the data was performed by JC, HT and RB. JC, RK and RL produced the first draft of the manuscript, with all other authors providing critical review and intellectual content. All authors read and approved the final manuscript.

Acknowledgements

We are very grateful to all the parents who took part in the research interviews. The views expressed in this publication are those of the authors and not necessarily any of the funding bodies listed.

For peer review only

References

1. World Health Organization. Coronavirus disease (COVID-19) pandemic [Internet]. 2020 [cited 2020 Nov 25]. Available from: https://www.who.int/emergencies/diseases/novel-coronavirus-2019

2. Public Health England. Health matters: physical activity - prevention and management of long-term conditions [Internet]. 2020 [cited 2020 Nov 30]. Available from: https://www.gov.uk/government/publications/health-matters-physical-activity/health-matters-physical-activity-prevention-and-management-of-long-term-conditions

3. Lawler R. New report shows scale of crisis in early years sector [Internet]. Early Years Alliance. 2020 [cited 2020 Nov 25]. Available from: https://www.eyalliance.org.uk/news/2020/06/new-report-shows-scale-crisis-early-years-sector

4. Wu Z, McGoogan JM. Characteristics of and Important Lessons from the Coronavirus Disease 2019 (COVID-19) Outbreak in China: Summary of a Report of 72314 Cases from the Chinese Center for Disease Control and Prevention [Internet]. JAMA - Journal of the American Medical Association. American Medical Association; 2020 [cited 2020 Nov 23]. p. 1239–42. Available from: https://jamanetwork.com/

5. Department of Health and Social Care. Physical activity guidelines: UK Chief Medical Officers' report [Internet]. [cited 2020 Nov 30]. Available from: https://www.gov.uk/government/collections/physical-activity-guidelines

6. www.nhs.co.uk. How much sleep do children need? - NHS [Internet]. 2020 [cited 2020 Nov 24]. Available from: https://www.nhs.uk/live-well/sleep-and-tiredness/how-much-sleep-do-kids-need/

For peer review only

7. Hesketh KR, Griffin SJ, van Sluijs EMF. UK Preschool-aged children’s physical activity levels in childcare and at home: A cross-sectional exploration. International Journal of Behavioral Nutrition and Physical Activity [Internet]. BioMed Central Ltd.; 2015 [cited 2020 Nov 24];12:123. Available from: https://ijbnpa.biomedcentral.com/articles/10.1186/s12966-015-0286-1

8. Brazendale K, Beets MW, Weaver RG, Pate RR, Turner-McGrievy GM, Kaczynski AT, et al. Understanding differences between summer vs. school obesogenic behaviors of children: The structured days hypothesis. International Journal of Behavioral Nutrition and Physical Activity [Internet]. BioMed Central Ltd.; 2017 [cited 2020 Nov 24];14:1–14. Available from: https://link.springer.com/articles/10.1186/s12966-017-0555-2

9. Terry Clark. 31% of shoppers have changed their primary supermarket in lockdown. https://www.retail-focus.co.uk/31-of-shoppers-have-changed-their-primary-supermarket-in-lockdown/. 2020.

10. Royal College of Paediatrics and Child Health C& YPE team. COVID-19 - research studies on children and young people’s views. https://www.rcpch.ac.uk/resources/covid-19-research-studies-children-young-peoples-views. 2020.

11. Dodd H, Westbrook J, Lawrence P. Co-SPYCE STUDY - 1ST UPDATE [Internet]. https://emergingminds.org.uk/co-spyce-study-1st-update/. 2020 [cited 2020 Nov 24]. Available from: https://emergingminds.org.uk/co-spyce-study-1st-update/

12. Public Health Scotland. COVID-19 Early years resilience and impact survey (CEYRIS) [Internet]. 2020 [cited 2020 Nov 25]. Available from: http://www.healthscotland.scot/publications/covid-19-early-years-resilience-and-impact-survey-ceyris

13. Serdula MK, Ivery D, Coates RJ, Freedman DS, Williamson DF, Byers T. Do Obese Children Become Obese Adults? A Review of the Literature. Preventive Medicine [Internet]. 1993 [cited 2020 Nov 25];22:167–77. Available from: https://linkinghub.elsevier.com/retrieve/pii/S0091743583710145

14. National Child Measurement Programme, England 2018/19 School Year [Internet]. NHS Digital. [cited 2020 Nov 25]. Available from: https://digital.nhs.uk/data-and-information/publications/statistical/national-child-measurement-programme/2018-19-school-year

For peer review only

15. Department for Education U. Coronavirus (COVID-19) attendance in educational and early years settings in England – summary of returns to 17 April 2020 [Internet]. GOV.UK. 2020 [cited 2020 Nov 25]. Available from: https://assets.publishing.service.gov.uk/government/uploads/system/uploads/attachment_data/file/883173/COVID-19_attendance_in_education_settings_170420.pdf

16. Prime H, Wade M, Browne DT. Risk and resilience in family well-being during the COVID-19 pandemic. American Psychologist [Internet]. American Psychological Association Inc.; 2020 [cited 2020 Nov 25];75:631–43. Available from: http://doi.apa.org/getdoi.cfm?doi=10.1037/amp0000660

17. NAP SACC (UK): Nutrition and physical activity self-assessment for childcare [Internet]. [cited 2020 Nov 30]. Available from: https://www.isrctn.com/ISRCTN33134697

18. Guest G, MacQueen KM, Namey EE. Applied Thematic Analysis. Los Angeles, London, New Delhi, Singapore, Washington DC: Sage Publishing; 2012.

19. Hesketh KR, Lakshman R, van Sluijs EMF. Barriers and facilitators to young children's physical activity and sedentary behaviour: a systematic review and synthesis of qualitative literature. Obesity Reviews [Internet]. Blackwell Publishing Ltd; 2017 [cited 2020 Nov 25];18:987–1017. Available from: http://doi.wiley.com/10.1111/obr.12562

20. Physical activity guidelines: UK Chief Medical Officers' report - GOV.UK [Internet]. [cited 2020 Nov 25]. Available from: https://www.gov.uk/government/publications/physical-activity-guidelines-uk-chief-medical-officers-report

21. Moore SA, Faulkner G, Rhodes RE, Brussoni M, Chulak-Bozzer T, Ferguson LJ, et al. Impact of the COVID-19 virus outbreak on movement and play behaviours of Canadian children and youth: A national survey. International Journal of Behavioral Nutrition and Physical Activity [Internet]. BioMed Central; 2020 [cited 2020 Nov 25];17:85. Available from: https://ijbnpa.biomedcentral.com/articles/10.1186/s12966-020-00987-8

22. Zecevic CA, Tremblay L, Lovsin T, Michel L. Parental Influence on Young Children's Physical Activity. International Journal of Pediatrics. Hindawi Limited; 2010;2010:1–9.

For peer review only

23. Gardner B, Lally P, Wardle J. Making health habitual: The psychology of "habit-formation" and general practice [Internet]. British Journal of General Practice. British Journal of General Practice; 2012 [cited 2020 Nov 30]. p. 664–6. Available from: http://www.dh.gov.uk/prod_consum_dh/

24. Lally P, van Jaarsveld CHM, Potts HWW, Wardle J. How are habits formed: Modelling habit formation in the real world. European Journal of Social Psychology [Internet]. John Wiley & Sons, Ltd; 2010 [cited 2020 Nov 30];40:998–1009. Available from: https://onlinelibrary.wiley.com/doi/full/10.1002/ejsp.674

25. Andrew A, Cattan S, Costa-Dias M, Farquharson C, Kraftman L, Krutikova S, et al. Institute for Fiscal Studies: Family time use and home learning during the COVID-19 lockdown (IFS Report R178) [Internet]. 2020 [cited 2020 Nov 25]. Available from: http://www.ifs.org.uk/

26. Rundle AG, Park Y, Herbstman JB, Kinsey EW, Wang YC. COVID-19–Related School Closings and Risk of Weight Gain Among Children. Obesity. Blackwell Publishing Inc.; 2020. p. 1008–9.

27. Public Health England. National Child Measurement Programme 2020: Information for schools [Internet]. 2020 Nov. Available from: https://assets.publishing.service.gov.uk/government/uploads/system/uploads/attachment_data/file/931777/NCMP_operational_guidance_ADDENDUM_2020.pdf

28. Sinha Consultant I, Bennett Researcher D, Taylor-Robinson DC. They must not be left behind in its aftermath. [cited 2020 Nov 25]; Available from: https://www.

29. Hefferon C, Taylor C, Bennett D, Falconer C, Calmpbell M, Williams J, et al. Pre-publication: Priorities for the child public health response to the COVID-19 pandemic recovery in England. Archives of Disease in Childhood (in press). 2020;

30. Daniel Thomas. Unemployment rate hits highest level in three years [Internet]. BBC News. 2020 [cited 2020 Nov 25]. Available from: https://www.bbc.co.uk/news/business-54520521

31. Sweet O, Long J, Evans L, Lindley L, Welch G, Barlow J, et al. State of the Nation: Understanding Public Attitudes to The Early Years [Internet]. 2020 Nov. Available from: https://mk0royalfoundatcnhl0.kinstacdn.com/wp-content/uploads/2020/11/Ipsos-MORI-SON_report_FINAL_V2.4.pdf

For peer review only

32. Education Endowment Foundation. Impact of Covid-19 on primary school starters | Projects | Education Endowment Foundation | EEF [Internet]. 2020 [cited 2020 Nov 25]. Available from: https://educationendowmentfoundation.org.uk/projects-and-evaluation/projects/the-impact-of-covid-19-on-primary-school-starters/?utm_source=site&utm_medium=search&utm_campaign=site_search&search_term=impact%20of%20covid

For peer review only

Table 1.

Topic guide used by interviewers to guide discussions during semi-structured interviews with parents of pre-school children

Domain	Questions
Physical activity, sedentary behaviours and sleep	• What activities has your child been doing during the coronavirus disruption?<br>• During the time since lock-down started in March has your child been going out of your home and garden (if they have one)?<br>• Do you think your child is currently more or less active than before the disruption?<br>• What sitting activities has your child been doing during the coronavirus disruption? (e.g. screen time, educational activities, TV, games, social interaction)<br>• Do you think the changes since March due to coronavirus have affected your child's sleep?
Eating behaviours	• Have there been any changes in what your child has been eating because of the coronavirus disruption?<br>• Have there been any changes to how you get your food?<br>• Have there been any changes to how you prepare your food at home?<br>• Overall, do you think that your child is eating more or less healthily than before the disruption?

Table 2

Demographic characteristics of the parents of pre-school children who participated in a semi-structured interview (n=20)

Characteristic	Categories	N(%)
**Gender**	*Female*	16 (80)
	Male	4 (20)
**Age Group**	*21-25*	1 (5)
	26-30	7 (35)
	31-35	5 (25)
	36-40	2 (10)
	41-45	5 (25)
**Ethnicity**	*White*	12 (60)
	Asian or Asian British	4 (20)
	Black or Black British	1 (5)
	Mixed	3 (15)
**IMD Quintile** (1 = most deprived)	*1*	10 (50)
	2	2 (10)
	3	3 (15)
	4	3 (15)
	5	2 (10)
**Anyone in the household in paid employment**	*Yes*	17 (85)
	No	3 (15)
**Educated to degree level**	*Yes*	13 (65)
	No	7 (35)
**Single parent**	*Yes*	5 (25)
	No	15 (75)

For peer review only - http://bmjopen.bmj.com/site/about/guidelines.xhtml

BMJ Open

Impact of COVID-19 restrictions on pre-school children's eating, activity and sleep behaviours: a qualitative study

Journal:	*BMJ Open*
Manuscript ID	bmjopen-2021-051497.R1
Article Type:	Original research
Date Submitted by the Author:	15-Jul-2021
Complete List of Authors:	Clarke, Joanne; University of Birmingham, Institute of Applied Health Research<br>Kipping, Ruth; University of Bristol, Centre for Public Health, Population Health Sciences, Bristol Medical School<br>Chambers, Stephanie; University of Glasgow, School of Social and Political Sciences and MRC/CSO Social and Public Health Sciences Unit, Institute of Health and Wellbeing<br>Willis, Kate; University of Bristol, Centre for Public Health, Population Health Sciences, Bristol Medical School<br>Taylor, Hilary; University of Bristol, Centre for Public Health, Population Health Sciences, Bristol Medical School<br>Brophy, Rachel; University of Bristol, Centre for Public Health, Population Health Sciences, Bristol Medical School<br>Hannam, Kimberly; University of Bristol, Centre for Public Health, Population Health Sciences, Bristol Medical School<br>Simpson, Sharon; University of Glasgow, School of Social and Political Sciences and MRC/CSO Social and Public Health Sciences Unit, Institute of Health and Wellbeing<br>Langford, Rebecca; University of Bristol, Centre for Public Health, Population Health Sciences, Bristol Medical School
**Primary Subject Heading**:	Public health
Secondary Subject Heading:	Qualitative research
Keywords:	COVID-19, NUTRITION & DIETETICS, QUALITATIVE RESEARCH, PAEDIATRICS, PUBLIC HEALTH

SCHOLARONE™
Manuscripts

I, the Submitting Author has the right to grant and does grant on behalf of all authors of the Work (as defined in the below author licence), an exclusive licence and/or a non-exclusive licence for contributions from authors who are: i) UK Crown employees; ii) where BMJ has agreed a CC-BY licence shall apply, and/or iii) in accordance with the terms applicable for US Federal Government officers or employees acting as part of their official duties; on a worldwide, perpetual, irrevocable, royalty-free basis to BMJ Publishing Group Ltd ("BMJ") its licensees and where the relevant Journal is co-owned by BMJ to the co-owners of the Journal, to publish the Work in this journal and any other BMJ products and to exploit all rights, as set out in our licence.

The Submitting Author accepts and understands that any supply made under these terms is made by BMJ to the Submitting Author unless you are acting as an employee on behalf of your employer or a postgraduate student of an affiliated institution which is paying any applicable article publishing charge ("APC") for Open Access articles. Where the Submitting Author wishes to make the Work available on an Open Access basis (and intends to pay the relevant APC), the terms of reuse of such Open Access shall be governed by a Creative Commons licence – details of these licences and which Creative Commons licence will apply to this Work are set out in our licence referred to above.

Other than as permitted in any relevant BMJ Author's Self Archiving Policies, I confirm this Work has not been accepted for publication elsewhere, is not being considered for publication elsewhere and does not duplicate material already published. I confirm all authors consent to publication of this Work and authorise the granting of this licence.

For peer review only

Impact of COVID-19 restrictions on pre-school children's eating, activity and sleep behaviours: a qualitative study

Authors:

Clarke JL[1], Kipping R[2], Chambers S[3], Willis K[2], Taylor H[2], Brophy R[2], Hannam K[2], Simpson SA[4] Langford R[2]*

[1] Institute of Applied Health Research, University of Birmingham

[2] Centre for Public Health, Population Health Sciences, Bristol Medical School, University of Bristol

[3] School of Social and Political Sciences and MRC/CSO Social and Public Health Sciences Unit, Institute of Health and Wellbeing, University of Glasgow

[4] MRC/CSO Social and Public Health Sciences Unit, Institute of Health and Wellbeing, University of Glasgow

*corresponding author: beki.langford@bristol.ac.uk, Canynge Hall, 39 Whatley Road, Bristol, BS8 2PL, UK.

Abstract

Objectives: In spring 2020, the first COVID-19 national lockdown placed unprecedented restrictions on the behaviour and movements of the UK population. Citizens were ordered to 'stay at home', only allowed to leave their houses to buy essential supplies, attend medical appointments or exercise once a day. We explored how lockdown and its subsequent easing changed young children's everyday activities, eating and sleep habits to gain insight into the impact for health and wellbeing.

Design: In-depth qualitative interviews; data analysed using thematic analysis.

Setting: South West and West Midlands of England.

For peer review only - http://bmjopen.bmj.com/site/about/guidelines.xhtml

UOB Confidential & Sensitive

Participants: Twenty parents (16 mothers; 4 fathers) of pre-school age children (3-5 years) due to start school in September 2020. Half the sample were from Black, Asian or Minority Ethnic backgrounds and half lived in the most deprived areas.

Results: Children's activity, screen time, eating, and sleep routines had been disrupted. Parents reported children ate more snacks, but families also spent more time preparing meals and eating together. Most parents reported a reduction in their children's physical activity and an increase in screen time, which some linked to difficulties in getting their child to sleep. Parents sometimes expressed guilt about changes in activity, screen time and snacking over lockdown. Most felt these changes would be temporary, though others worried about re-establishing healthy routines.

Conclusions: Parents reported that lockdown negatively impacted on pre-school children's eating, activity and sleep routines. While some positive changes were reported, many participants reported of lack of routines, habits and boundaries which may have been detrimental for child health and development. Guidance and support for families during COVID-19 restrictions could be valuable to help maintain healthy activity, eating, screen-time and sleeping routines to protect child health and ensure unhealthy habits are not adopted.

Strengths and Limitations of this study

- Few studies have explored the perceived impact of COVID-19 restrictions on young children or health behaviours like physical activity or diet.
- Our in-depth interviews provide novel insights into the perceived impact of lockdown restrictions on pre-school children’s physical activity, sedentary behaviour, food intake and sleep.

For peer review only - http://bmjopen.bmj.com/site/about/guidelines.xhtml
UOB Confidential & Sensitive

- A strength of the study was the ethnic and socioeconomic diversity of the sample with half of participants from the lowest deprivation quintile and half from Black, Asian or Minority ethnicities.
- Interviews were conducted with parents, rather than preschool children themselves, for pragmatic reasons.

Key words

COVID-19; coronavirus; pre-school; child; paediatric; physical activity; sleep; qualitative; diet; screen time

For peer review only

For peer review only - http://bmjopen.bmj.com/site/about/guidelines.xhtml

UOB Confidential & Sensitive

Background

The emergence of COVID-19, and subsequent efforts to restrict its spread, led to unprecedented economic and social disruption[1]. Many countries imposed restrictions on citizens' behaviours and movements to limit the spread of the virus, including social distancing and local or national 'lockdowns'.

The UK Government imposed its first 'lockdown' in March 2020. Citizens were required to 'stay at home' only leaving to buy essential supplies, attend medical appointments or to exercise. Schools and nurseries were closed (except to vulnerable or 'keyworker' children), as were leisure facilities, pubs, restaurants, theatres and non-essential shops. Non-essential workplaces were closed, and people could not meet anyone beyond their immediate household. Unlike more stringent lockdowns elsewhere, UK citizens were permitted to exercise outside once a day[2]. Restrictions lasted over 2 months until late May 2020. Pre-schools reopened to all children in June 2020, alongside schools who re-opened to certain year groups at this point; however, occupancy remained low with attendance half that recorded for the previous year (37% vs.77%) [3].

Despite being least affected by the virus[4]children have experienced enormous disruption which may have impacted their health behaviours. Children under five should have 180 minutes of physical activity a day [5]. Children aged 3-4 should have 11-13 hours sleep in a 24-hour period [6]. With the closure of pre-schools, lockdown reduced children's opportunities for physical activity, while increasing the likelihood of sedentary behaviours [7] and had the potential to disrupt sleep patterns [8]. The spring 2020 lockdown also changed people's food shopping and eating habits. Consumers moved to less frequent shopping trips, but spent more on food than pre-lockdown, potentially changing the home food environment [9].

Establishing healthy behaviours in the early years is important in maintaining a healthy weight [10]. Nearly one in four UK children starting primary school are overweight or obese, with rates increasing with deprivation[11]. Many children saw time in childcare reduced or stop altogether [12]. Pre-school children

For peer review only - http://bmjopen.bmj.com/site/about/guidelines.xhtml

UOB Confidential & Sensitive

For peer review only

require greater parental supervision which may mean managing children's activities and behaviours in lockdown was more challenging for these families, particularly if parents were working or there were additional family stressors [13].

Several surveys have sought to capture the impact of COVID-19 on children and young people's health and well-being. Most have focused on emotional wellbeing and have often targeted older children[14]. Less attention has focused on younger (pre-school) children or explored the impact on physical activity, sedentary behaviour and diet. An exception is the Co-SPYCE study [15] which surveyed caregivers of 2-4 year olds. It found 26% of children had 3+ hours of screen time a day, and only 22% were active for 180 minutes/day. Public Health Scotland developed the COVID-19 Early Years Resilience and Impact Survey (CEYRIS) [16] for 2-7 year-olds in Scotland. From 11,228 participants, eating behaviour was identified as worse by 32% of parents. There were mixed responses about physical activity, with 24% reporting children doing more during lockdown and 47% reported less.

While these quantitative surveys provide a useful overview, few studies have used qualitative methods to understand in greater depth how pre-school children have been affected by these restrictions. To address this gap, we used in-depth interviews with parents of 3-5 year-olds to explore the impact of the first UK COVID-19 lockdown on pre-school children's eating, activity and sleep behaviours.

Methods

Population, sampling and recruitment

Criteria for inclusion were: (1) parent/carer of a child aged 3-5 years in their final year at pre-school; (2) child usually attends pre-school at least once per week; (3) child due to start school in September. Non-English speakers were excluded for pragmatic reasons.

Parents were recruited via two methods. First, we asked nursery staff participating in another research study [17] to disseminate study information to parents via email or social media. This included nurseries from Swindon and Somerset (South West England) and Sandwell (West Midlands). Second, we posted study information on local Facebook groups within these areas and Birmingham (West Midlands) to increase recruitment of Black, Asian and Minority Ethnic (BAME) participants. Interested parents (n=85)

For peer review only - http://bmjopen.bmj.com/site/about/guidelines.xhtml

UOB Confidential & Sensitive

For peer review only

completed a form to check eligibility and collect postcode (deprivation) and ethnicity data for sampling purposes. To provide diversity, sampling was weighted towards parents in the most deprived areas and those from BAME populations. Twelve parents were recruited via nurseries and eight via Facebook.

Sampled parents were emailed the participant information sheet (PIS) and consent form. Interviews were arranged with participants at least 24 hours after sending study documents. All sampled parents agreed to participate, bar one who we were subsequently unable to contact.

Patient and public involvement

Due to time constraints, we did not directly involve parents or children in the planning of this study. However, nursery staff provided advice on the best ways to recruit parents for this study.

Data collection

Semi-structured interviews were conducted by experienced qualitative researchers (JC, KW or SC) in July/August 2020 by telephone (n=18) or video (n=2). Before interview, the researcher explained the study aims, answered questions and gained consent. A topic guide (Table 1, see also supplementary materials) was used to guide discussions with parents encouraged to talk openly about their experiences. Interviews were audio-recorded, transcribed verbatim and anonymised. Following each interview, notes were made of key points raised. A £30 shopping voucher was sent to participants following interview.

Data analysis

Data were analysed thematically [18] using NVivo (version 12) to aid data management and analysis. All transcripts were read by JC, RB and HT to gain familiarity with the data. Three transcripts were independently coded by these researchers to generate an initial list of codes. Codes were both deductive (generated from topic guide and research questions) and inductive (generated from interview data). Differences in coding were resolved through discussion to produce an agreed coding framework. Subsequent transcripts were single-coded using this framework with further discussion to clarify or expand the framework as needed.

For peer review only - http://bmjopen.bmj.com/site/about/guidelines.xhtml

UOB Confidential & Sensitive

Ethical approval

Ethical approval was granted by University of Bristol Faculty of Health Sciences Research Ethics Committee (ref: 106002).

Results

Participants

Sixteen mothers and four fathers participated in the study (Table 2). Average age of parents was 34 years (range 21-45 years). Half the sample were White British. Thirteen participants were educated to degree level. Half resided in the most deprived Index of Multiple Deprivation (IMD) quintile. Five participants were single parents. Thirteen reported at least one parent was not working during the lockdown period; three reported that no household member was currently employed. Two participants had no access to a garden. Despite the variation within our sample, we found few differences in the reported impact of lockdown across the socioeconomic spectrum, or by other participant characteristics.

Food and eating

Increased snacking

Most parents reported their child's snacking had increased over lockdown, which they often linked to stay-at-home restrictions. Children were bored and consequently snacked more: "*When there's nothing to occupy them, they've got to do something"* (Mother_1). Some felt loss of routine had disrupted rules around snacking: *"if he's at pre-school he can't snack. He can only eat at the specific snack time…Here, he just thinks it's on tap."* (Mother_2). Some found dealing with demands for snacks challenging, with some parents permitting more "unhealthy" items than normal: *"It's all we hear. She's constantly hungry… She's definitely eating more bags of crisps than she normally would"* (Mother_3).

Some parents created make-shift rules around food, balancing child demands with wanting to provide "healthy" snacks. Mother_4's children were allowed crisps because they went *"on and on"* but then were told *"to move on to the fruit bowl."* Another parent made healthy snacks more accessible: "*I*

For peer review only - http://bmjopen.bmj.com/site/about/guidelines.xhtml

UOB Confidential & Sensitive

For peer review only

make sure there are bits and bobs cut up in the fridge… If she's going to help herself to something, it's the stuff on the bottom she can reach" (Mother_5). However, some parents gave additional 'treats' to their children to compensate for the pandemic's impact: *"We were feeling bad they'd missed out on so much, we were buying treats to make them feel happy."* (Mother_6)

Food preparation and mealtimes

Some parents reported an increase in problematic mealtime behaviours, such as children not wanting to eat or taking a long time to eat. Often this was linked to increased snacking. However, several reported positive effects on mealtime routines. With parents working from home, families could eat together more often. Similarly, parents enjoyed having time to cook and involve their child. This helped occupy their child and taught them new skills: "*When we couldn't go to McDonald's we made homemade chicken nuggets… that's been posi*tive, *they've enjoyed cooking"* (Mother_6).

Lockdown had other impacts on families' food behaviours. With restaurants shut, people no longer ate out. Some participants also reported consuming less take-aways. For one, this was related to concerns about the virus: "*I didn't touch any takeaways. I didn't feel comfortable." (*Mother_4). In contrast, one parent reported eating more take-aways because "w*e all need something to look forward to"* (Mother_7).

Food costs

Several participants reported spending more on food during lockdown. Some related this to the whole family being at home, as well as increased snacking, while others felt prices increased. One family no longer had access to free meals at their child's nursery. Another participant who was shielding had moved to on-line deliveries, a service unavailable at their normal cheaper supermarkets: "*We'd normally just go to Aldi or Lidl… now we've been trying to do the online stuff, it's just added to our bill*" (Mother_9). However, an unexpected benefit of this was healthier snacking: "*it's a money thing, we've not been able to buy all the junk food*" (Mother_9).

For peer review only - http://bmjopen.bmj.com/site/about/guidelines.xhtml
UOB Confidential & Sensitive

Physical activity

Most parents felt their child was less active during lockdown. For some this was substantial - *"we* [were] *nowhere near as active as we used to be"* (Mother_4) – while a minority felt it had little impact. Several related this reduction to childcare closures which removed usual opportunities for activity*: "I put it down to routine and being less active… At nursery, you're on the go from the time you get there to the time you go home"* (Mother_10). Opportunities for active transport to and from childcare were also lost: *"[She's] definitely less active because we walk to [pre-]school and that was 10 minutes there and 10 minutes back… She's not having that exercise*" (Mother_11). Normal activities (like swimming, soft play) were also closed and opportunities to socialise with others restricted.

Parents noted several factors that helped children stay active. Having a sibling was important, providing someone to play with. Equally, access to play equipment helped facilitate activity: *"They've got a trampoline, a bike each, a scooter each"* (Mother_4). Access to outside space was also important. Only two parents had no access to outdoor space, but both described this as challenging during lockdown. As Mother_8 explained, "*We couldn't leave and there was nothing really you could do. And living in a block of flats, you can't be too noisy because you've got neighbours everywhere*" (Mother_8). In contrast, Mother_1 noted how having a safe space to cycle outside her house helped keep her son active: "*My front* [garden] *is really nice for children to play and ride bikes."*

Good weather was also seen as an important facilitator of activity: "*We had the pool out and [he] was using it quite a lot … We were pretty much burning energy off most of the time, especially with the nice weather we had*" (Mother_12). Some parents discussed how they made use of the local environment during lockdown: *"We've done a lot of exploring around the local area… we've found some places we didn't know were there"* (Mother_6).

Parents often put effort into keeping their child physically active: *"I've tried really hard to make sure he's been as active as possible"* (Mother_7). This was easier for families where one parent was not

For peer review only - http://bmjopen.bmj.com/site/about/guidelines.xhtml

UOB Confidential & Sensitive

working: "*He has to have a parent with him. 'Look at me! Look at my star jumps!'… That was the difficult bit, because there is only so much of that you can do, especially when you've got work.*" (Father_1)

Sedentary behaviours

Parents reported their child engaged in a range of sedentary activities during lockdown (like reading, drawing) but screen time was mentioned most frequently with almost all parents reporting substantial increases. For many families, screen time filled the void left by being unable to go out or socialise. Mother_13 explained they watched more television because "*we were stuck in the house… we weren't having that release of getting out",* while Mother_1 commented "[My son] *can't go to friends, he can't meet family… so the screen had become his friend".*

Many felt screen time had been useful during this difficult period, with one parent referring to it as a *"lifesaver"* (Father_1). For those trying to work from home, it was often the only way they could manage: "*If I've got a two-hour Zoom meeting and I don't want him bursting in every five seconds I'd give him his iPad and he'll watch a film*" (Mother_6). Even for those not working, managing everyday chores was a challenge: *"You've still got housework to do, washing to do, meals to prepare… You can't always be there 24/7 to keep her occupied"* (Mother_3).

Screen time was also used to provide much needed respite from the intensive parenting effort lockdown enforced. Father_2 described screen time as providing "*a break*" while Mother_11 used it to avoid constant sibling squabbles: *"They're just following you around … fighting and hitting each other… just sit down quietly and watch a movie."* However, parents' language suggested many felt uncomfortable about increased screen time. Mother_6 admitted *"It's bad, I don't even like saying it out loud, but [screen time] was almost like a pacifier, really*", while Mother_3 admitted "*It's going to sound horrible, but it's just sometimes easier".*

Parents often distinguished between 'good' and 'bad' screen time. Educational or interactive screen time was better than passive television watching. Mother_11 would ask herself "*Is it educational or is it*

For peer review only - http://bmjopen.bmj.com/site/about/guidelines.xhtml

UOB Confidential & Sensitive

For peer review only

just rubbish they're watching?", while Mother_3 explained "*I don't want her to mindlessly sit there watching a screen. At least if she's playing [educational] games, it's making her think.*"

Concerns over screen time were rarely linked to lack of movement or activity. Rather Father_3 referred to *"the effect of the brain becoming lazy."* Mother_5 suggested too much screen time interfered with normal childhood and fostered an inability to occupy oneself: "*They're not being children. I suppose you think about your own childhood, don't you? All the bits and bobs I used to do as a kid. When I've said no screen time, they just wander around like aimless sheep.*"

Some parents created new rules to manage increased screen time. This included restrictions on the amount, times of day or content. Mother_14 explained, "*We have had to impose rules… we've just had to structure it a bit more, because she would gladly sit in front of the telly or iPad all day.*" Other families took a more reactive approach: "*We're not a routine sort of family anyway, so if they've had an hour or so, then I'll be like, 'Right, that's it now, everybody out.'*" (Mother_5)

Sleep

Almost half of parents reported negative changes to their child's sleep. Many reported difficulties in getting their child to sleep, with some staying up very late. Parents often related this either to lack of activity – "*it's because she's not been as active and been out*" (Mother_8) – or increased screen time – "*a direct correlation of being a bit more bored, spending more time on a screen*" (Mother_3). Loss of routine was again a source of difficulty for some families. As Mother_4 commented: "*Before it was a very strict* [bedtime] *routine ... now it is kind of as and when*". A minority of parents wondered if anxiety played a part in their child's sleep difficulties: "*I'm just wondering whether* [his frequent waking] *was a part of maybe anxiety or worried about everything that's going on.*" (Mother_12)

Longer term impacts

Generally, parents thought children's increase in snacking during lockdown was "*just a temporary thing*" (Mother_2). As Mother_5 acknowledged: "*when she goes to school she's not going to have that*

For peer review only - http://bmjopen.bmj.com/site/about/guidelines.xhtml
UOB Confidential & Sensitive

opportunity to (a) steal food or (b) she won't be home to be eating masses during the day". However, one parent whose child had already returned to nursery acknowledged getting back into a routine was difficult: *"Trying to get him to eat properly once we started to go back into a routine was really hard"* (Mother_10).

Several parents wished to sustain positive changes established during lockdown, such as home-cooked meals and involving children in food preparation. However, these changes required time and would need a *"conscious effort to keep doing"* (Mother_14). Many talked positively about returning to previous activities (e.g. swimming, gymnastics) though several admitted feeling *"wary"* (Father_4) about how safe these were, especially those involving close contact.

Some parents were concerned about the impact of lockdown on their child's skills and confidence. Mother_14 felt her daughter had lost her swimming skills and noted a general loss of confidence: "*She's a lot more cautious on her bike because for a while they just didn't leave the house… she's had a step backwards with that.*" Another was concerned about lost stamina and wondered if her daughter would manage the mile-long walk to school: "*I am actually a little bit worried about how she's going to fare… she's not been used to doing that walk*" (Mother_5).

Most parents were not overly concerned about increased screen time, assuming it would naturally decrease with a return to routine. However, others wondered how an increased reliance on screens during lockdown might change their child's behaviours going forwards. Mother_6 described her son pre-lockdown as a *"pocket rocket*" who never sat still. Now, however, "*rather than going outside and running around the garden, as soon as he wakes up [he asks] 'Can I have the iPad?'…He would quite happily sit all morning.*" Similarly, Mother_3 explained her daughter would now *"rather have something on the telly than go out".* Allowing her daughter to watch television in her mother's bedroom during lockdown, Mother_3 acknowledged the difficulty in re-establishing pre-lockdown boundaries: "*She's asking Santa for a television for her bedroom, which I'm hoping she forgets.*"

For peer review only - http://bmjopen.bmj.com/site/about/guidelines.xhtml

UOB Confidential & Sensitive

Discussion

During the first COVID-19 lockdown parents reported increased snacking, screen time and sleep issues, and decreased physical activity in their pre-school children. There were also positive family behaviour changes, such as more eating together and use of the local environment for physical activity. Some parents expressed concerns about the future impact of negative behaviour changes, but most believed these were a temporary consequence of a lack of routine.

This study provides in-depth reports of family life with a pre-school child during the pandemic, and complements findings of parents of 2-4 year olds from the Co-SPYCE survey [15] which revealed children's screen time as a key parental stressor during lockdown. Parental use of children's screen time to 'get things done' was a key finding of a recent qualitative systematic review [19]. In our study, with the absence of childcare, and corresponding increase in parental juggling of work and household chores, it is clear how this 'need' for downtime increased for many.

Most parents felt their child was less active over lockdown echoing findings from other studies. Only 22% of 2-4 year olds in the Co-SPYCE study were reported as getting 180 minutes of activity per day [5] and almost half (47%) of Scottish parents reported their children (2-7yrs) did less physical activity during lockdown [16]. A Canadian survey of parents of young children (18m-5yrs) reported half of children were less physically active and 87% were engaged in more screen time during the initial COVID-19 restrictions compared to pre-pandemic[20]. Survey of older children in the US and Canada found children were less active and engaged in more screen time during the initial COVID-19 outbreak compared with before restrictions,[21,22]. Parent encouragement, participation and support of physical activity were found to be important correlates of children's physical activity [23]. Zecevic et al [24] showed that, in circumstances not associated with lockdown, young children who receive parental support for physical activity were more likely to be highly active than inactive. In our study some parents made considerable efforts to keep their child active, using the opportunity to leave the house once per day for physical activity to explore the local environment (in contrast to restrictions in other countries such as Spain where the

For peer review only - http://bmjopen.bmj.com/site/about/guidelines.xhtml
UOB Confidential & Sensitive

population was not permitted to leave home for exercise [25]). Yet despite these parental efforts, barriers specific to lockdown were reported which reduced activity, mainly relating to the closure of childcare and playgrounds, and reduced contact with friends and family.

Sleep disruptions reported by parents in our study echo findings of a questionnaire study with mothers of Italian pre-schoolers where more challenging bedtime routines and decreased sleep quality were reported as an early impact of lockdown [26].

Our study identified disruptions to normal eating habits and routines including changes in snacking behaviour, frequency and volume of eating and who children were eating with. While some families speculated these changes would soon return to normal, it may be difficult to re-establish boundaries and break new habits [27,28]. One parent discussed how free meals for her child at nursery had ceased. While our study did not identify acute food poverty, it is likely that pre-school children will have experienced food poverty where free school meals were no longer available and where the pandemic impacted family finances [29].

Rundle et al (2020) suggested the closure of schools during this period may exacerbate childhood obesity levels[30]. While few parents mentioned weight, our findings suggest there is potential for obesity prevalence in pre-school children to have increased due to increased snacking and screen time, and decreased physical activity, particularly if changes during lockdown have persisted. The annual National Child Measurement Programme was stopped in March 2020 but restarted in January 2021. This will enable assessment of the impact on children who started school in September 2020 [31].

The negative impacts of COVID-19 are expected to be greatest for children living in poverty[32,33]We sought to explore differences in the impact of lockdown across the socioeconomic spectrum. Half our participants lived in the most deprived IMD quintile, giving insight into those living in the poorest areas. We noted some differences within our sample. Two families without access to gardens reflected on the difficulty in keeping children active. Families where parents were not working found it

For peer review only - http://bmjopen.bmj.com/site/about/guidelines.xhtml

UOB Confidential & Sensitive

easier to engage children in physical activity. Beyond this, however, we did not note any obvious differences between those living in more or less deprived neighbourhoods. The impact of lockdown on children's behaviours was felt similarly across the socioeconomic spectrum. The lack of difference could be due to sample characteristics; most families still had at least one parent working and access to a garden. Interviews took place early in the COVID-19 crisis, so we may not yet be seeing the full effect, for example with the protective effect of the furlough scheme still in place.

This study raises questions about how parents can be supported during future lockdowns or local restrictions. Subsequent UK lockdowns in late 2020 and early 2021 presented new and additional challenges: shorter, colder days, weariness of the restrictions, and increased financial pressures with high rates of unemployment and redundancy[34]. In addition, positive cases in childcare or school settings can result in children being required to isolate for 10 days without being allowed out. The importance of supporting families to maintain or increase activity for young children within the home and in accessible outdoor environments becomes more prominent. Families need support to establish healthy revised routines and manageable healthy rules for snacks and screen time during periods of restriction. This must be presented in a context of supporting parents and not adding guilt or burden to parents during a period of stress. A recent Ipsos Mori report, which included an online survey of 1,000 parents of 0-5s, found 70% of parents felt judged by others and almost half felt this negatively impacted on their mental health [35]. There is a role here for all sectors of health, social care and education including health visitors, children's centres, early years providers, GPs, social services, local authority public health teams and Public Health England. Further, there is a role for research with pre-schoolers and 2020 school starters to be expanded [36] to understand the impact on their physical and social development, and particularly their risk of obesity, as they start school.

Strengths and limitations

In-depth interviews enabled us to gain novel insights into the experiences of parents and pre-school children during lockdown and restrictions, though we acknowledge the limitation of investigating pre-

For peer review only - http://bmjopen.bmj.com/site/about/guidelines.xhtml

UOB Confidential & Sensitive

school children's experiences by asking their parents, rather than involving pre-school children directly. A strength of the study was the ethnic and socioeconomic diversity of the sample from two regions of England, although this was a relatively small (n=20) and educated sample. Parents volunteering for interview may have had more interest in the topic or had different experiences to those who did not volunteer. We acknowledge that the use of remote interviews could have compromised participants' ability to express themselves and non-verbal clues may have been missed[37]. Other primary caregivers were eligible for the study, but none were recruited. Rigour was achieved by detailed data analysis and analytical decisions being shared with all team members to achieve credibility.

Conclusions

While some positive changes were reported, there were wide-spread examples of lack of routines, habits and boundaries which, at least in the short-term, were likely to have been detrimental for child health and development. Guidance and support for parents and families on how to maintain healthy routines and compensate for future COVID-19 restrictions could be valuable to protect child health and ensure that unhealthy habits are not adopted.

For peer review only

For peer review only - http://bmjopen.bmj.com/site/about/guidelines.xhtml
UOB Confidential & Sensitive

Licence statement

I, the Submitting Author has the right to grant and does grant on behalf of all authors of the Work (as defined in the below author licence), an exclusive licence and/or a non-exclusive licence for contributions from authors who are: i) UK Crown employees; ii) where BMJ has agreed a CC-BY licence shall apply, and/or iii) in accordance with the terms applicable for US Federal Government officers or employees acting as part of their official duties; on a worldwide, perpetual, irrevocable, royalty-free basis to BMJ Publishing Group Ltd ("BMJ") its licensees and where the relevant Journal is co-owned by BMJ to the co-owners of the Journal, to publish the Work in BMJ Open and any other BMJ products and to exploit all rights, as set out in our licence.

Abbreviations

BAME	Black, Asian and Minority Ethnic
CO-SPYCE	Covid-19: Supporting Parents and Young Children during Epidemics (study)
COVID-19	Coronavirus Disease (also known as SARS-CoV-2)
GPs	General Practitioners
IMD	Index of Multiple Deprivation
UK	United Kingdom

Ethics approval and consent to participate

Ethical approval for the study was granted by University of Bristol Faculty of Health Sciences Research Ethics Committee. Participant consent was recorded on a separate audio file before the interview commenced.

For peer review only - http://bmjopen.bmj.com/site/about/guidelines.xhtml

UOB Confidential & Sensitive

For peer review only

Consent for publication

Consent to include anonymised information in publications was given by all participants.

Availability of data and materials

Anonymised study data will be made available via a University of Bristol repository.

Competing interests

The authors declare that they have no competing interests.

Funding

This work was funded by the NIHR School for Public Health Research (SPHR-PROG-CYP-WP3) and NIHR funding for the NAP SACC UK trial (2019-3426).

Authors' contributions

The study was conceived by JC, RK, SC, KW, HT, RB, KH, SS and RL. JC led the study with oversight from RL and RK. Interviews were conducted by JC, SC and KW. Coding of the data was performed by JC, HT and RB. JC, RK and RL produced the first draft of the manuscript, with all other authors providing critical review and intellectual content. All authors read and approved the final manuscript.

Acknowledgements

We are very grateful to all the parents who took part in the research interviews. The views expressed in this publication are those of the authors and not necessarily any of the funding bodies listed.

For peer review only

For peer review only - http://bmjopen.bmj.com/site/about/guidelines.xhtml
UOB Confidential & Sensitive

References

1 World Health Organization. Coronavirus disease (COVID-19) pandemic. 2020.https://www.who.int/emergencies/diseases/novel-coronavirus-2019 (accessed 7 Jul 2021).

2 Public Health England. Health matters: physical activity - prevention and management of long-term conditions. 2020. https://www.gov.uk/government/publications/health-matters-physical-activity/health-matters-physical-activity-prevention-and-management-of-long-term-conditions (accessed 7 Jul 2021).

3 Lawler R. New report shows scale of crisis in early years sector. Early Years Alliance. 2020. https://www.eyalliance.org.uk/news/2020/06/new-report-shows-scale-crisis-early-years-sector (accessed 7 Jul 2021).

4 Wu Z, McGoogan JM. Characteristics of and Important Lessons from the Coronavirus Disease 2019 (COVID-19) Outbreak in China: Summary of a Report of 72314 Cases from the Chinese Center for Disease Control and Prevention. *JAMA*. 2020;**323**:1239–42. doi:10.1001/jama.2020.2648

5 Foster C, Reilly J, Jago R, *et al.* UK Chief Medical Officers' Physical Activity Guidelines. 2019. https://assets.publishing.service.gov.uk/government/uploads/system/uploads/attachment_data/file/832868/uk-chief-medical-officers-physical-activity-guidelines.pdf (accessed 7 Jul 2021).

6 NHS UK. How much sleep do children need? 2017.https://www.nhs.uk/live-well/sleep-and-tiredness/how-much-sleep-do-kids-need/ (accessed 7 Jul 2021).

7 Hesketh KR, Griffin SJ, van Sluijs EMF. UK Preschool-aged children's physical activity levels in childcare and at home: A cross-sectional exploration. *International Journal of Behavioral Nutrition and Physical Activity* 2015;**12**:123. doi:10.1186/s12966-015-0286-1

8 Brazendale K, Beets MW, Weaver RG, *et al.* Understanding differences between summer vs. school obesogenic behaviors of children: The structured days hypothesis. *International Journal of Behavioral Nutrition and Physical Activity* 2017;**14**:1–14. doi:10.1186/s12966-017-0555-2

9 Clark T. 31% of shoppers have changed their primary supermarket in lockdown. https://www.retail-focus.co.uk/31-of-shoppers-have-changed-their-primary-supermarket-in-lockdown/. (accessed 7 Jul 2021).

For peer review only - http://bmjopen.bmj.com/site/about/guidelines.xhtml

UOB Confidential & Sensitive

10 Serdula MK, Ivery D, Coates RJ, *et al.* Do Obese Children Become Obese Adults? A Review of the Literature. *Preventive Medicine* 1993;**22**:167–77. doi:10.1006/pmed.1993.1014

11 NHS Digital. National Child Measurement Programme, England 2018/19 School Year. https://digital.nhs.uk/data-and-information/publications/statistical/national-child-measurement-programme/2018-19-school-year (accessed 7 Jul 2021).

12 Department for Education. Coronavirus (COVID-19) attendance in educational and early years settings in England – summary of returns to 17 April 2020. GOV.UK. 2020. https://assets.publishing.service.gov.uk/government/uploads/system/uploads/attachment_data/file/883173/COVID-19_attendance_in_education_settings_170420.pdf (accessed 7 Jul 2021).

13 Prime H, Wade M, Browne DT. Risk and resilience in family well-being during the COVID-19 pandemic. *American Psychologist* 2020;**75**:631–43. doi:10.1037/amp0000660

14 Royal College of Paediatrics and Child Health, Children & Young People's Engagement team. COVID-19 - research studies on children and young people's views. 2020. https://www.rcpch.ac.uk/resources/covid-19-research-studies-children-young-peoples-views (accessed 7 Jul 2021).

15 Dodd H, Westbrook J, Lawrence P. Co-SPYCE STUDY - 1ST UPDATE. https://emergingminds.org.uk/co-spyce-study-1st-update/. 2020. https://emergingminds.org.uk/co-spyce-study-1st-update/ (accessed 7 Jul 2021).

16 Public Health Scotland. COVID-19 Early years resilience and impact survey (CEYRIS). 2020. http://www.healthscotland.scot/publications/covid-19-early-years-resilience-and-impact-survey-ceyris (accessed 7 Jul 2021).

17 NAP SACC (UK): Nutrition and physical activity self-assessment for childcare. 2019. https://www.isrctn.com/ISRCTN33134697 (accessed 7 Jul 2021).

18 Guest G, MacQueen KM, Namey EE. *Applied Thematic Analysis*. Los Angeles, London, New Delhi, Singapore, Washington DC: Sage Publishing 2012.

For peer review only - http://bmjopen.bmj.com/site/about/guidelines.xhtml

UOB Confidential & Sensitive

19 Hesketh KR, Lakshman R, van Sluijs EMF. Barriers and facilitators to young children's physical activity and sedentary behaviour: a systematic review and synthesis of qualitative literature. *Obesity Reviews* 2017;**18**:987–1017. doi:10.1111/obr.12562

20 Carroll N, Sadowski A, Laila A, *et al.* The Impact of COVID-19 on Health Behavior, Stress, Financial and Food Security among Middle to High Income Canadian Families with Young Children. *Nutrients* 2020;**8**. doi:10.3390/nu12082352

21 Dunton GF, Do B, Wang SD. Early effects of the COVID-19 pandemic on physical activity and sedentary behavior in children living in the U.S. *BMC Public Health* 2020;**20**:1–13. doi:10.1186/s12889-020-09429-3

22 Moore SA, Faulkner G, Rhodes RE, *et al.* Impact of the COVID-19 virus outbreak on movement and play behaviours of Canadian children and youth: A national survey. *International Journal of Behavioral Nutrition and Physical Activity* 2020;**17**:1–11. doi:10.1186/s12966-020-00987-8

23 Moore SA, Faulkner G, Rhodes RE, *et al.* Impact of the COVID-19 virus outbreak on movement and play behaviours of Canadian children and youth: A national survey. *International Journal of Behavioral Nutrition and Physical Activity* 2020;**17**:85. doi:10.1186/s12966-020-00987-8

24 Zecevic CA, Tremblay L, Lovsin T, *et al.* Parental Influence on Young Children's Physical Activity. *International Journal of Pediatrics* 2010;**2010**:1–9. doi:10.1155/2010/468526

25 Idoiaga Mondragon N, Berasategi Sancho N, Dosil Santamaria M, *et al.* Struggling to breathe: a qualitative study of children's wellbeing during lockdown in Spain. *Psychology and Health* 2021;**36**:179–94. doi:10.1080/08870446.2020.1804570

26 Dellagiulia A, Lionetti F, Fasolo M, *et al.* Early impact of COVID-19 lockdown on children's sleep: A 4-week longitudinal study. *Journal of Clinical Sleep Medicine*. 2020;**16**:1639–40. doi:10.5664/jcsm.8648

27 Gardner B, Lally P, Wardle J. Making health habitual: The psychology of "habit-formation" and general practice. *British Journal of General Practice*. 2012;**62**:664–6. doi:10.3399/bjgp12X659466

28 Lally P, van Jaarsveld CHM, Potts HWW, *et al.* How are habits formed: Modelling habit formation in the real world. *European Journal of Social Psychology* 2010;**40**:998–1009. doi:10.1002/ejsp.674

For peer review only - http://bmjopen.bmj.com/site/about/guidelines.xhtml
UOB Confidential & Sensitive

29 Andrew A, Cattan S, Costa-Dias M, *et al.* Family time use and home learning during the COVID-19 lockdown. 2020. https://ifs.org.uk/publications/15038 (accessed 7 Jul 2021).

30 Rundle AG, Park Y, Herbstman JB, *et al.* COVID-19–Related School Closings and Risk of Weight Gain Among Children. *Obesity*. 2020;**28**:1008–9. doi:10.1002/oby.22813

31 Public Health England. National Child Measurement Programme 2020: Information for schools. 2020. https://assets.publishing.service.gov.uk/government/uploads/system/uploads/attachment_data/file/931777/NCMP_operational_guidance_ADDENDUM_2020.pdf (accessed 7 Jul 2021).

32 Sinha I, Bennett D, Taylor-Robinson DC. Children are being sidelined by covid-19. *BMJ* 2020;**369**. doi:10.1136/BMJ.M2061

33 Hefferon C, Taylor C, Bennett D, *et al.* Priorities for the child public health response to the COVID-19 pandemic recovery in England. *Archives of disease in childhood* 2021;**106**. doi:10.1136/ARCHDISCHILD-2020-320214

34 Thomas D. Unemployment rate hits highest level in three years. BBC News. 2020. https://www.bbc.co.uk/news/business-54520521 (accessed 7 Jul 2021).

35 Sweet O, Long J, Evans L, *et al.* State of the Nation: Understanding Public Attitudes to The Early Years. 2020. https://mk0royalfoundatcnhl0.kinstacdn.com/wp-content/uploads/2020/11/Ipsos-MORI-SON_report_FINAL_V2.4.pdf (accessed 7 Jul 2021).

36 Education Endowment Foundation. Impact of Covid-19 on primary school starters. 2020. https://educationendowmentfoundation.org.uk/projects-and-evaluation/projects/the-impact-of-covid-19-on-school-starters/ (accessed 7 Jul 2021).

37 Clarke V, Braun V. *Successful Qualitative Research: A Practical Guide for Beginners*. 1st ed. London: Sage 2013.

For peer review only

For peer review only - http://bmjopen.bmj.com/site/about/guidelines.xhtml

UOB Confidential & Sensitive

Table 1

Summary of topic guide used by interviewers to guide discussions during semi-structured interviews with parents of pre-school children. The full topic guide is available as supplementary material. This table was created by the co-authors for this publication.

Interview topics
Physical activity<br>– *Impact on activity in and out of house. More/less/same as before? Why?*
Sedentary activities<br>– *Impact on and types of activities. More/less/same as before? Why?*
Sleep behaviours<br>– *Impact on bedtimes, getting to sleep and staying asleep. More/less/same as before? Why?*
Eating<br>– *Impact on what and when food is eaten (mealtimes & snacks). More/less/ same as before? Why?*
Food purchasing<br>– *Amount, type, cost and access to food*
Food preparation<br>– *Changes to who, how, when food is prepared and why*

For peer review only

For peer review only - http://bmjopen.bmj.com/site/about/guidelines.xhtml
UOB Confidential & Sensitive

Table 2

Demographic characteristics of the parents of pre-school children who participated in a semi-structured interview (n=20). This table was created by the co-authors for this publication.

**Characteristic Categories**		**N(%)**
**Gender**	*Female*	16 (80)
	Male	4 (20)
**Age Group**	*21-25*	1 (5)
	26-30	7 (35)
	31-35	5 (25)
	36-40	2 (10)
	41-45	5 (25)
**Ethnicity**	*White*	12 (60)
	Asian or Asian British	4 (20)
	Black or Black British	1 (5)
	Mixed	3 (15)
**IMD Quintile** (1 = most deprived)	*1*	10 (50)
	2	2 (10)
	3	3 (15)
	4	3 (15)
	5	2 (10)
**Anyone in the household in paid employment**	*Yes*	17 (85)
	No	3 (15)
**Educated to degree level**	*Yes*	13 (65)
	No	7 (35)
**Single parent**	*Yes*	5 (25)
	No	15 (75)

For peer review only

For peer review only - http://bmjopen.bmj.com/site/about/guidelines.xhtml
UOB Confidential & Sensitive

Coronavirus Preschool Study: a qualitative study of the lived experience of final year pre-school children in the UK during the coronavirus (COVID-19) disruption – Interview Topic Guide

Introduction

- Hello [name], it's [name] from the University of Bristol/Birmingham/Glasgow speaking, how are you doing? Are you still ok to chat to me today? It will take up to an hour.
- Thank you so much for sparing your time to speak to me today.
- Before starting the interview, I'm going to talk a bit about the study. I'm then going to take your consent before we start the interview.
- Firstly, just to give a bit of background of the study, we want to find out about how pre-school children are doing during the coronavirus disruption, and what effects, if any, it is having on their mood and their physical activity and eating behaviours. Throughout the interview I will refer to coronavirus which some people call COVID-19.
- When answering the questions, please try to focus on your final year pre-school child rather than any other children in the household.
- We are interested in the whole period since mid-March, i.e. lockdown, easing of restrictions and now.
- Feel free to ask questions at any time.
- There are no wrong or right answers and you do not have to answer all of the questions if you do not want to.
- Anything that you tell me is confidential and will not be linked to you. The only reason for me needing to break confidentiality would be if you say something where I am concerned about harm to you or someone else.
- You can stop at any time and if you decide in the next two weeks that you do not want me to include what you said, just let me know.
- I have already sent you a copy of the consent form along with the participant information sheet. When I start the voice recorder, I'm just going to read out each statement on the consent form one by one and if you could please confirm that you're happy with each statement. This is something we have to do as part of the University ethics regulations.
- Do you have any questions before I start the recording? If it's ok, I will start recording now.

START RECORDING

- State interviewer name, participant name, participant ID number and date/time of interview.
- Read out each statement on the consent form. Ask participant to say 'Yes' after each statement if they are happy with the statement.
- Explain to the participant that you are now going to stop the audio-recording, and re-start recording for the main part of the interview.

STOP RECORDING

START RECORDING

State interviewer name, participant ID and date/time of interview (do NOT state participant name).

For peer review only - http://bmjopen.bmj.com/site/about/guidelines.xhtml

Background

Tell me about your household	• Name/age/gender of child focusing on for this interview<br>• Relationship of interviewee to child<br>• Age/gender of any other children<br>• Do you have a garden?
Is anyone in the house working ?	• Any keyworkers ?<br>• Anyone working from home; furloughed?
Has anyone in the household had coronavirus ?	• Tested or untested

Childcare

Remind participant that the focus of the interview is on the final year preschool child (not on other children in the family).

**Before** the coronavirus disruption, what were you doing for childcare?	• Setting (e.g. nursery, childminder, family members)<br>• How many days/hours per week of childcare?
**During** the lockdown when nurseries were shut except to key workers, did your child attend any childcare?	• If so, why? As much as before, or less?
How have you been managing childcare with the coronavirus disruption?	• Who is/has been looking after the children?<br>• If your nursery has **re-opened**, is your child currently attending any childcare? Is this as more or less than before?<br>• If your child has not returned to childcare, can you tell me why?<br>• If you child has returned to childcare, what changes have been made in childcare because of coronavirus? (e.g. attending part-time, parents not allowed in, smaller groups, changes to food provision)

Your child's mood and how they have been feeling

note to interviewer – we are interested in the whole period since mid-March, i.e. lockdown, easing of restrictions and now

How has your child been feeling about the coronavirus restrictions?	• What effects (if any) do you think the restrictions have had on your child's well-being?<br>• Have the restrictions had any effect on your child's mood and feelings? (positive or negative)<br>• Have you noticed any changes at all in your child's behaviour? (positive or negative)<br>• What is the impact on the child of any reduced social contact with other children, friends and family?

For peer review only

For peer review only - http://bmjopen.bmj.com/site/about/guidelines.xhtml

Physical activity

We're interesting in how the lockdown and the social distancing restrictions that are happening now might have affected young children's activities they do when they're moving around or sat still. By moving around, I mean anything from: Pottering (e.g. Lego, helping around the house, small-world play, dressing up); On the Go: (e.g. playing in the garden, dancing, hide and seek, playing on the furniture, rough and tumble); and Huff and Puff: (e.g. running games, trampolining, scooting, dancing).

note to interviewer – we are interested in the whole period since mid-March, i.e. lockdown, easing of restrictions and now

What activities has your child been doing during the coronavirus disruption?	• Has the coronavirus disruption had any effect on how active your child is?<br>• What has changed? What has stopped?
During the time since lock-down started in March has your child been going out of your home and garden (if they have one)?	• If not, why not?<br>• Has this changed as lockdown has been eased?
Do you think your child is currently more or less active than before the disruption?	• Why?<br>• How do you think any changes will affect how active your child is in the future?<br>• Will you re-start any activities that you can no longer do?<br>• Will you carry on doing any new activities?
What sitting activities has your child been doing during the coronavirus disruption? (e.g. screen time, educational activities, TV, games, social interaction)	• Has there been any change in the amount of time the child spends sitting?<br>• Have there been any changes in screen time rules or supervision of screen time in your house during the disruption?<br>• How do you think any changes will affect future screen use?
Do you think the changes since March due to coronavirus have affected your child's sleep?	• In what way?

School

How are you feeling about your child starting school in September?	• How is your child feeling?<br>• Any thoughts about whether child will start in September, or whether you may delay starting school?
Are there any things you would have been doing to help prepare the child for starting school that you aren't able to do because of the disruption?	• E.g. visits to new school, end of preschool special events<br>• What could help with the transition during this time of uncertainty?

Nutrition

note to interviewer – we are interested in the whole period since mid-March, i.e. lockdown, easing of restrictions and now

For peer review only - http://bmjopen.bmj.com/site/about/guidelines.xhtml

Have there been any changes in what your child has been eating because of the coronavirus disruption?	• E.g. changes in quantity of food, types of food?<br>• Any changes in how you get your food, what food you can get, how much you spend?<br>• Changes in snacking/treats?<br>• Any changes to food preparation at home? E.g. more/fewer take away meals, cooking with child, cooking from scratch?<br>• Changes in fruit and vegetable consumption?
Overall, do you think that your child is eating more or less healthily than before the disruption?	• Why?<br>• Thinking about the future, do you think that any changes to your child's eating will continue?

Demographic questions

- We would like to ask you some questions about you and your children. If you do not wish to answer, please just say so.
- If you don't mind telling me, what is your age?
- How would you describe your ethnicity?
- Out of the following options, what is your current employment status?
  - Student
  - Stay at home parent/caregiver
  - Full-time
  - Part-time
  - Unemployed
  - Furloughed
- What is your highest level of education? Options:
  - Up to GCSEs/GCEs/O levels or similar
  - A levels/NVQs/GNVQs
  - First degree/diploma/HNC/HND
  - Higher degree (e.g. MSc, PhD)
- Finally, how did you find out about this research study i.e. where did you see the study advert?

End

- That's all the questions I have for you today. Do you have any questions for me?
- I will stop recording the interview now.

STOP RECORDING

Thank the participant and explain that a voucher will be emailed to them within 7 days.

For peer review only - http://bmjopen.bmj.com/site/about/guidelines.xhtml
For peer review only

Impact of COVID-19 restrictions on pre-school children's eating, activity and sleep behaviours: a qualitative study

Journal:	*BMJ Open*
Manuscript ID	bmjopen-2021-051497.R2
Article Type:	Original research
Date Submitted by the Author:	02-Sep-2021
Complete List of Authors:	Clarke, Joanne; University of Birmingham, Institute of Applied Health Research<br>Kipping, Ruth; University of Bristol, Centre for Public Health, Population Health Sciences, Bristol Medical School<br>Chambers, Stephanie; University of Glasgow, School of Social and Political Sciences and MRC/CSO Social and Public Health Sciences Unit, Institute of Health and Wellbeing<br>Willis, Kate; University of Bristol, Centre for Public Health, Population Health Sciences, Bristol Medical School<br>Taylor, Hilary; University of Bristol, Centre for Public Health, Population Health Sciences, Bristol Medical School<br>Brophy, Rachel; University of Bristol, Centre for Public Health, Population Health Sciences, Bristol Medical School<br>Hannam, Kimberly; University of Bristol, Centre for Public Health, Population Health Sciences, Bristol Medical School<br>Simpson, Sharon; University of Glasgow, School of Social and Political Sciences and MRC/CSO Social and Public Health Sciences Unit, Institute of Health and Wellbeing<br>Langford, Rebecca; University of Bristol, Centre for Public Health, Population Health Sciences, Bristol Medical School
**Primary Subject Heading**:	Public health
Secondary Subject Heading:	Qualitative research
Keywords:	COVID-19, NUTRITION & DIETETICS, QUALITATIVE RESEARCH, PAEDIATRICS, PUBLIC HEALTH

SCHOLARONE™ Manuscripts

I, the Submitting Author has the right to grant and does grant on behalf of all authors of the Work (as defined in the below author licence), an exclusive licence and/or a non-exclusive licence for contributions from authors who are: i) UK Crown employees; ii) where BMJ has agreed a CC-BY licence shall apply, and/or iii) in accordance with the terms applicable for US Federal Government officers or employees acting as part of their official duties; on a worldwide, perpetual, irrevocable, royalty-free basis to BMJ Publishing Group Ltd ("BMJ") its licensees and where the relevant Journal is co-owned by BMJ to the co-owners of the Journal, to publish the Work in this journal and any other BMJ products and to exploit all rights, as set out in our licence.

The Submitting Author accepts and understands that any supply made under these terms is made by BMJ to the Submitting Author unless you are acting as an employee on behalf of your employer or a postgraduate student of an affiliated institution which is paying any applicable article publishing charge ("APC") for Open Access articles. Where the Submitting Author wishes to make the Work available on an Open Access basis (and intends to pay the relevant APC), the terms of reuse of such Open Access shall be governed by a Creative Commons licence – details of these licences and which Creative Commons licence will apply to this Work are set out in our licence referred to above.

Other than as permitted in any relevant BMJ Author's Self Archiving Policies, I confirm this Work has not been accepted for publication elsewhere, is not being considered for publication elsewhere and does not duplicate material already published. I confirm all authors consent to publication of this Work and authorise the granting of this licence.

For peer review only

Impact of COVID-19 restrictions on pre-school children's eating, activity and sleep behaviours: a qualitative study

Authors:

Clarke JL[1], Kipping R[2], Chambers S[3], Willis K[2], Taylor H[2], Brophy R[2], Hannam K[2], Simpson SA[4] Langford R[2]*

[1] Institute of Applied Health Research, University of Birmingham

[2] Centre for Public Health, Population Health Sciences, Bristol Medical School, University of Bristol

[3] School of Social and Political Sciences and MRC/CSO Social and Public Health Sciences Unit, Institute of Health and Wellbeing, University of Glasgow

[4] MRC/CSO Social and Public Health Sciences Unit, Institute of Health and Wellbeing, University of Glasgow

*corresponding author: beki.langford@bristol.ac.uk, Canynge Hall, 39 Whatley Road, Bristol, BS8 2PL, UK.

Abstract

Objectives: In spring 2020, the first COVID-19 national lockdown placed unprecedented restrictions on the behaviour and movements of the UK population. Citizens were ordered to 'stay at home', only allowed to leave their houses to buy essential supplies, attend medical appointments or exercise once a day. We explored how lockdown and its subsequent easing changed young children's everyday activities, eating and sleep habits to gain insight into the impact for health and wellbeing.

Design: In-depth qualitative interviews; data analysed using thematic analysis.

Setting: South West and West Midlands of England.

For peer review only - http://bmjopen.bmj.com/site/about/guidelines.xhtml
UOB Confidential & Sensitive
For peer review only

Participants: Twenty parents (16 mothers; 4 fathers) of pre-school age children (3-5 years) due to start school in September 2020. Half the sample were from Black, Asian or Minority Ethnic backgrounds and half lived in the most deprived areas.

Results: Children's activity, screen time, eating, and sleep routines had been disrupted. Parents reported children ate more snacks, but families also spent more time preparing meals and eating together. Most parents reported a reduction in their children's physical activity and an increase in screen time, which some linked to difficulties in getting their child to sleep. Parents sometimes expressed guilt about changes in activity, screen time and snacking over lockdown. Most felt these changes would be temporary, though others worried about re-establishing healthy routines.

Conclusions: Parents reported that lockdown negatively impacted on pre-school children's eating, activity and sleep routines. While some positive changes were identified, many participants described lack of routines, habits and boundaries which may have been detrimental for child health and development. Guidance and support for families during COVID-19 restrictions could be valuable to help maintain healthy activity, eating, screen-time and sleeping routines to protect child health and ensure unhealthy habits are not adopted.

Strengths and Limitations of this study

- Few studies have explored the perceived impact of COVID-19 restrictions on young children or health behaviours like physical activity or diet.
- Our in-depth interviews provide novel insights into the perceived impact of lockdown restrictions on pre-school children’s physical activity, sedentary behaviour, food intake and sleep.

For peer review only - http://bmjopen.bmj.com/site/about/guidelines.xhtml

UOB Confidential & Sensitive

- A strength of the study was the ethnic and socioeconomic diversity of the sample with half of participants from the lowest deprivation quintile and half from Black, Asian or Minority ethnicities.
- Interviews were conducted with parents, rather than preschool children themselves, for pragmatic reasons.

Key words

COVID-19; coronavirus; pre-school; child; paediatric; physical activity; sleep; qualitative; diet; screen time

For peer review only

For peer review only - http://bmjopen.bmj.com/site/about/guidelines.xhtml

UOB Confidential & Sensitive

Background

The emergence of COVID-19, and subsequent efforts to restrict its spread, led to unprecedented economic and social disruption[1]. Many countries imposed restrictions on citizens' behaviours and movements to limit the spread of the virus, including social distancing and local or national 'lockdowns'.

The UK Government imposed its first 'lockdown' in March 2020. Citizens were required to 'stay at home' only leaving to buy essential supplies, attend medical appointments or to exercise. Schools and nurseries were closed (except to vulnerable or 'keyworker' children), as were leisure facilities, pubs, restaurants, theatres and non-essential shops. Non-essential workplaces were closed, and people could not meet anyone beyond their immediate household. Unlike more stringent lockdowns elsewhere, UK citizens were permitted to exercise outside once a day[2]. Restrictions lasted over 2 months until late May 2020. Pre-schools reopened to all children in June 2020, alongside schools who re-opened to certain year groups at this point; however, occupancy remained low with attendance half that recorded for the previous year (37% vs.77%) [3].

Despite being least affected by the virus[4] children have experienced enormous disruption which may have impacted their health behaviours. Children under five should have 180 minutes of physical activity a day [5]. Children aged 3-4 should have 11-13 hours sleep in a 24-hour period [6]. The closure of pre-schools, playgrounds and the 'stay at home' order during lockdown reduced children's opportunities for physical activity, increased the likelihood of sedentary behaviours [7] and had the potential to disrupt sleep patterns [8]. The spring 2020 lockdown also changed people's food shopping and eating habits. Consumers moved to less frequent shopping trips, but spent more on food than pre-lockdown, potentially changing the home food environment [9].

Establishing healthy behaviours in the early years is important in maintaining a healthy weight [10]. Nearly one in four UK children starting primary school are overweight or obese, with rates increasing with deprivation[11]. Many children saw time in childcare reduced or stop altogether [12]. Pre-school children

For peer review only - http://bmjopen.bmj.com/site/about/guidelines.xhtml
UOB Confidential & Sensitive

require greater parental supervision which may mean managing children's activities and behaviours in lockdown was more challenging for these families, particularly if parents were working or there were additional family stressors [13].

Several surveys have sought to capture the impact of COVID-19 on children and young people's health and well-being. Most have focused on emotional wellbeing and have often targeted older children[14]. Less attention has focused on younger (pre-school) children or explored the impact on physical activity, sedentary behaviour and diet. An exception is the Co-SPYCE study [15] which surveyed caregivers of 2-4 year olds. It found 26% of children had 3+ hours of screen time a day, and only 22% were active for 180 minutes/day. Public Health Scotland developed the COVID-19 Early Years Resilience and Impact Survey (CEYRIS) [16] for 2-7 year-olds in Scotland. From 11,228 participants, eating behaviour was identified as worse by 32% of parents. There were mixed responses about physical activity, with 24% reporting children doing more during lockdown and 47% reported less.

While these quantitative surveys provide a useful overview, few studies have used qualitative methods to understand in greater depth how pre-school children have been affected by these restrictions. To address this gap, we used in-depth interviews with parents of 3-5 year-olds to explore the impact of the first UK COVID-19 lockdown on pre-school children's eating, activity and sleep behaviours.

Methods

Population, sampling and recruitment

Criteria for inclusion were: (1) parent/carer of a child aged 3-5 years in their final year at pre-school; (2) child usually attends pre-school at least once per week; (3) child due to start school in September. Non-English speakers were excluded for pragmatic reasons.

Parents were recruited via two methods. First, we asked nursery staff participating in another research study [17] to disseminate study information to parents via email or social media. This included nurseries from Swindon and Somerset (South West England) and Sandwell (West Midlands). Second, we posted study information on local Facebook groups within these areas and Birmingham (West Midlands) to increase recruitment of Black, Asian and Minority Ethnic (BAME) participants. Interested parents (n=85)

For peer review only - http://bmjopen.bmj.com/site/about/guidelines.xhtml

UOB Confidential & Sensitive

For peer review only

completed a form to check eligibility and collect postcode (deprivation) and ethnicity data for sampling purposes. To provide diversity, sampling was weighted towards parents in the most deprived areas and those from BAME populations. Twelve parents were recruited via nurseries and eight via Facebook.

Sampled parents were emailed the participant information sheet (PIS) and consent form. Interviews were arranged with participants at least 24 hours after sending study documents. All sampled parents agreed to participate, bar one who we were subsequently unable to contact.

Patient and public involvement

Due to time constraints, we did not directly involve parents or children in the planning of this study. However, nursery staff provided advice on the best ways to recruit parents for this study.

Data collection

Semi-structured interviews were conducted by experienced qualitative researchers (JC, KW or SC) in July/August 2020 by telephone (n=18) or video (n=2). Before interview, the researcher explained the study aims, answered questions and gained consent. A topic guide (Table 1, see also supplementary materials) was used to guide discussions with parents encouraged to talk openly about their experiences. Interviews were audio-recorded, transcribed verbatim and anonymised. Following each interview, notes were made of key points raised. A £30 shopping voucher was sent to participants following interview.

Data analysis

Data were analysed thematically [18] using NVivo (version 12) to aid data management and analysis. All transcripts were read by JC, RB and HT to gain familiarity with the data. Three transcripts were independently coded by these researchers to generate an initial list of codes. Codes were both deductive (generated from topic guide and research questions) and inductive (generated from interview data). Differences in coding were resolved through discussion to produce an agreed coding framework. Subsequent transcripts were single-coded using this framework with further discussion to clarify or expand the framework as needed.

For peer review only - http://bmjopen.bmj.com/site/about/guidelines.xhtml
UOB Confidential & Sensitive

Ethical approval

Ethical approval was granted by University of Bristol Faculty of Health Sciences Research Ethics Committee (ref: 106002).

Results

Participants

Sixteen mothers and four fathers participated in the study (Table 2). Average age of parents was 34 years (range 21-45 years). Half the sample were White British. Thirteen participants were educated to degree level. Half resided in the most deprived Index of Multiple Deprivation (IMD) quintile. Five participants were single parents. Thirteen reported at least one parent was not working during the lockdown period; three reported that no household member was currently employed. Two participants had no access to a garden. Despite the variation within our sample, we found few differences in the reported impact of lockdown across the socioeconomic spectrum, or by other participant characteristics.

Food and eating

Increased snacking

Most parents reported their child's snacking had increased over lockdown, which they often linked to stay-at-home restrictions. Children were bored and consequently snacked more: "*When there's nothing to occupy them, they've got to do something*" (Mother_1). Some felt loss of routine had disrupted rules around snacking: "*if he's at pre-school he can't snack. He can only eat at the specific snack time…Here, he just thinks it's on tap.*" (Mother_2). Some found dealing with demands for snacks challenging, with some parents permitting more "unhealthy" items than normal: *"It's all we hear. She's constantly hungry… She's definitely eating more bags of crisps than she normally would"* (Mother_3).

Some parents created make-shift rules around food, balancing child demands with wanting to provide "healthy" snacks. Mother_4's children were allowed crisps because they went *"on and on"* but then were told "*to move on to the fruit bowl.*" Another parent made healthy snacks more accessible: "*I*

For peer review only - http://bmjopen.bmj.com/site/about/guidelines.xhtml

UOB Confidential & Sensitive

make sure there are bits and bobs cut up in the fridge… If she's going to help herself to something, it's the stuff on the bottom she can reach" (Mother_5). However, some parents gave additional 'treats' to their children to compensate for the pandemic's impact: *"We were feeling bad they'd missed out on so much, we were buying treats to make them feel happy."* (Mother_6)

Food preparation and mealtimes

Some parents reported an increase in problematic mealtime behaviours, such as children not wanting to eat or taking a long time to eat. Often this was linked to increased snacking. However, several reported positive effects on mealtime routines. With parents working from home, families could eat together more often. Similarly, parents enjoyed having time to cook and involve their child. This helped occupy their child and taught them new skills: "*When we couldn't go to McDonald's we made homemade chicken nuggets… that's been posi*tive, *they've enjoyed cooking"* (Mother_6).

Lockdown had other impacts on families' food behaviours. With restaurants shut, people no longer ate out. Some participants also reported consuming less take-aways. For one, this was related to concerns about the virus: "*I didn't touch any takeaways. I didn't feel comfortable." (*Mother_4). In contrast, one parent reported eating more take-aways because "w*e all need something to look forward to"* (Mother_7).

Food costs

Several participants reported spending more on food during lockdown. Some related this to the whole family being at home, as well as increased snacking, while others felt prices increased. One family no longer had access to free meals at their child's nursery. Another participant who was shielding had moved to on-line deliveries, a service unavailable at their normal cheaper supermarkets: "*We'd normally just go to Aldi or Lidl… now we've been trying to do the online stuff, it's just added to our bill*" (Mother_9). However, an unexpected benefit of this was healthier snacking: "*it's a money thing, we've not been able to buy all the junk food*" (Mother_9).

For peer review only - http://bmjopen.bmj.com/site/about/guidelines.xhtml

UOB Confidential & Sensitive

Physical activity

Most parents felt their child was less active during lockdown. For some this was substantial - *"we* [were] *nowhere near as active as we used to be"* (Mother_4) – while a minority felt it had little impact. Several related this reduction to childcare closures which removed usual opportunities for activity*: "I put it down to routine and being less active… At nursery, you're on the go from the time you get there to the time you go home"* (Mother_10)*.* Opportunities for active transport to and from childcare were also lost: "*[She's] definitely less active because we walk to [pre-]school and that was 10 minutes there and 10 minutes back… She's not having that exercise*" (Mother_11). Normal activities (like swimming, soft play) were also closed and opportunities to socialise with others restricted.

Parents noted several factors that helped children stay active. Having a sibling was important, providing someone to play with. Equally, access to play equipment helped facilitate activity: *"They've got a trampoline, a bike each, a scooter each"* (Mother_4). Access to outside space was also important. Only two parents had no access to outdoor space, but both described this as challenging during lockdown. As Mother_8 explained, "*We couldn't leave and there was nothing really you could do. And living in a block of flats, you can't be too noisy because you've got neighbours everywhere*" (Mother_8). In contrast, Mother_1 noted how having a safe space to cycle outside her house helped keep her son active: "*My front* [garden] *is really nice for children to play and ride bikes.*"

Good weather was also seen as an important facilitator of activity: "*We had the pool out and [he] was using it quite a lot … We were pretty much burning energy off most of the time, especially with the nice weather we had*" (Mother_12). Some parents discussed how they made use of the local environment during lockdown: *"We've done a lot of exploring around the local area… we've found some places we didn't know were there"* (Mother_6).

Parents often put effort into keeping their child physically active: *"I've tried really hard to make sure he's been as active as possible"* (Mother_7). This was easier for families where one parent was not

For peer review only - http://bmjopen.bmj.com/site/about/guidelines.xhtml

UOB Confidential & Sensitive

working: "*He has to have a parent with him. 'Look at me! Look at my star jumps!'… That was the difficult bit, because there is only so much of that you can do, especially when you've got work.*" (Father_1)

Sedentary behaviours

Parents reported their child engaged in a range of sedentary activities during lockdown (like reading, drawing) but screen time was mentioned most frequently with almost all parents reporting substantial increases. For many families, screen time filled the void left by being unable to go out or socialise. Mother_13 explained they watched more television because "*we were stuck in the house… we weren't having that release of getting out",* while Mother_1 commented "[My son] *can't go to friends, he can't meet family… so the screen had become his friend".*

Many felt screen time had been useful during this difficult period, with one parent referring to it as a *"lifesaver"* (Father_1). For those trying to work from home, it was often the only way they could manage: "*If I've got a two-hour Zoom meeting and I don't want him bursting in every five seconds I'd give him his iPad and he'll watch a film*" (Mother_6). Even for those not working, managing everyday chores was a challenge: *"You've still got housework to do, washing to do, meals to prepare… You can't always be there 24/7 to keep her occupied"* (Mother_3).

Screen time was also used to provide much needed respite from the intensive parenting effort lockdown enforced. Father_2 described screen time as providing "*a break*" while Mother_11 used it to avoid constant sibling squabbles: *"They're just following you around … fighting and hitting each other… just sit down quietly and watch a movie."* However, parents' language suggested many felt uncomfortable about increased screen time. Mother_6 admitted *"It's bad, I don't even like saying it out loud, but [screen time] was almost like a pacifier, really*", while Mother_3 admitted "*It's going to sound horrible, but it's just sometimes easier".*

Parents often distinguished between 'good' and 'bad' screen time. Educational or interactive screen time was better than passive television watching. Mother_11 would ask herself "*Is it educational or is it*

For peer review only - http://bmjopen.bmj.com/site/about/guidelines.xhtml

UOB Confidential & Sensitive

just rubbish they're watching?", while Mother_3 explained "*I don't want her to mindlessly sit there watching a screen. At least if she's playing [educational] games, it's making her think.*"

Concerns over screen time were rarely linked to lack of movement or activity. Rather Father_3 referred to *"the effect of the brain becoming lazy."* Mother_5 suggested too much screen time interfered with normal childhood and fostered an inability to occupy oneself: "*They're not being children. I suppose you think about your own childhood, don't you? All the bits and bobs I used to do as a kid. When I've said no screen time, they just wander around like aimless sheep*."

Some parents created new rules to manage increased screen time. This included restrictions on the amount, times of day or content. Mother_14 explained, "*We have had to impose rules… we've just had to structure it a bit more, because she would gladly sit in front of the telly or iPad all day*." Other families took a more reactive approach: "*We're not a routine sort of family anyway, so if they've had an hour or so, then I'll be like, 'Right, that's it now, everybody out.'*" (Mother_5)

Sleep

Almost half of parents reported negative changes to their child's sleep. Many reported difficulties in getting their child to sleep, with some staying up very late. Parents often related this either to lack of activity – "*it's because she's not been as active and been out*" (Mother_8) – or increased screen time – "*a direct correlation of being a bit more bored, spending more time on a screen"* (Mother_3). Loss of routine was again a source of difficulty for some families. As Mother_4 commented: "*Before it was a very strict* [bedtime] *routine ... now it is kind of as and when"*. A minority of parents wondered if anxiety played a part in their child's sleep difficulties: "*I'm just wondering whether* [his frequent waking] *was a part of maybe anxiety or worried about everything that's going on*." (Mother_12)

Longer term impacts

Generally, parents thought children's increase in snacking during lockdown was "*just a temporary thing"* (Mother_2). As Mother_5 acknowledged: "*when she goes to school she's not going to have that*

For peer review only - http://bmjopen.bmj.com/site/about/guidelines.xhtml
UOB Confidential & Sensitive

opportunity to (a) steal food or (b) she won't be home to be eating masses during the day". However, one parent whose child had already returned to nursery acknowledged getting back into a routine was difficult: *"Trying to get him to eat properly once we started to go back into a routine was really hard"* (Mother_10).

Several parents wished to sustain positive changes established during lockdown, such as home-cooked meals and involving children in food preparation. However, these changes required time and would need a *"conscious effort to keep doing"* (Mother_14). Many talked positively about returning to previous activities (e.g. swimming, gymnastics) though several admitted feeling *"wary"* (Father_4) about how safe these were, especially those involving close contact.

Some parents were concerned about the impact of lockdown on their child's skills and confidence. Mother_14 felt her daughter had lost her swimming skills and noted a general loss of confidence: "*She's a lot more cautious on her bike because for a while they just didn't leave the house… she's had a step backwards with that*." Another was concerned about lost stamina and wondered if her daughter would manage the mile-long walk to school: "*I am actually a little bit worried about how she's going to fare… she's not been used to doing that walk*" (Mother_5).

Most parents were not overly concerned about increased screen time, assuming it would naturally decrease with a return to routine. However, others wondered how an increased reliance on screens during lockdown might change their child's behaviours going forwards. Mother_6 described her son pre-lockdown as a *"pocket rocket*" who never sat still. Now, however, "*rather than going outside and running around the garden, as soon as he wakes up [he asks] 'Can I have the iPad?'…He would quite happily sit all morning*." Similarly, Mother_3 explained her daughter would now *"rather have something on the telly than go out".* Allowing her daughter to watch television in her mother's bedroom during lockdown, Mother_3 acknowledged the difficulty in re-establishing pre-lockdown boundaries: "*She's asking Santa for a television for her bedroom, which I'm hoping she forgets.*"

For peer review only - http://bmjopen.bmj.com/site/about/guidelines.xhtml

UOB Confidential & Sensitive

Discussion

During the first COVID-19 lockdown parents reported increased snacking, screen time and sleep issues, and decreased physical activity in their pre-school children. There were also positive family behaviour changes, such as more eating together and use of the local environment for physical activity. Some parents expressed concerns about the future impact of negative behaviour changes, but most believed these were a temporary consequence of a lack of routine.

This study provides in-depth reports of family life with a pre-school child during the pandemic, and complements findings of parents of 2-4 year olds from the Co-SPYCE survey [15] which revealed children's screen time as a key parental stressor during lockdown. Parental use of children's screen time to 'get things done' was a key finding of a recent qualitative systematic review [19]. In our study, with the absence of childcare, and corresponding increase in parental juggling of work and household chores, it is clear how this 'need' for downtime increased for many.

Most parents felt their child was less active over lockdown echoing findings from other studies. Only 22% of 2-4 year olds in the Co-SPYCE study were reported as getting 180 minutes of activity per day [5] and almost half (47%) of Scottish parents reported their children (2-7yrs) did less physical activity during lockdown [16]. A Canadian survey of parents of young children (18m-5yrs) reported half of children were less physically active and 87% were engaged in more screen time during the initial COVID-19 restrictions compared to pre-pandemic[20]. Surveys of older children in the US and Canada found children were less active and engaged in more screen time during the initial COVID-19 outbreak compared with before restrictions,[21,22]. Parent encouragement, participation and support of physical activity were found to be important correlates of children's physical activity [23]. Zecevic et al [24] showed that, in circumstances not associated with lockdown, young children who receive parental support for physical activity were more likely to be highly active than inactive. In our study some parents made considerable efforts to keep their child active, using the opportunity to leave the house once per day for physical activity to explore the local environment (in contrast to restrictions in other countries such as Spain where the

For peer review only - http://bmjopen.bmj.com/site/about/guidelines.xhtml
UOB Confidential & Sensitive

population was not permitted to leave home for exercise [25]). Yet despite these parental efforts, barriers specific to lockdown were reported which reduced activity, mainly relating to the closure of childcare and playgrounds, and reduced contact with friends and family.

Sleep disruptions reported by parents in our study echo findings of a questionnaire study with mothers of Italian pre-schoolers where more challenging bedtime routines and decreased sleep quality were reported as an early impact of lockdown [26].

Our study identified disruptions to normal eating habits and routines including changes in snacking behaviour, frequency and volume of eating and who children were eating with. While some families speculated these changes would soon return to normal, it may be difficult to re-establish boundaries and break new habits [27,28]. One parent discussed how free meals for her child at nursery had ceased. While our study did not identify acute food poverty, it is likely that pre-school children will have experienced food poverty where free school meals were no longer available and where the pandemic impacted family finances [29].

For peer review only

Rundle et al (2020) suggested the closure of schools during this period may exacerbate childhood obesity levels[30]. While few parents mentioned weight, our findings suggest there is potential for obesity prevalence in pre-school children to have increased due to increased snacking and screen time, and decreased physical activity, particularly if changes during lockdown have persisted. The annual National Child Measurement Programme was stopped in March 2020 but restarted in January 2021. This will enable assessment of the impact on children who started school in September 2020 [31].

The negative impacts of COVID-19 are expected to be greatest for children living in poverty[32,33]We sought to explore differences in the impact of lockdown across the socioeconomic spectrum. Half our participants lived in the most deprived IMD quintile, giving insight into those living in the poorest areas. We noted some differences within our sample. Two families without access to gardens reflected on the difficulty in keeping children active. Families where parents were not working found it

For peer review only - http://bmjopen.bmj.com/site/about/guidelines.xhtml

UOB Confidential & Sensitive

easier to engage children in physical activity. Beyond this, however, we did not note any obvious differences between those living in more or less deprived neighbourhoods. The impact of lockdown on children's behaviours was felt similarly across the socioeconomic spectrum. The lack of difference could be due to sample characteristics; most families still had at least one parent working and access to a garden. Interviews took place early in the COVID-19 crisis, so we may not yet be seeing the full effect, for example with the protective effect of the furlough scheme still in place.

This study raises questions about how parents can be supported during future lockdowns or local restrictions. Subsequent UK lockdowns in late 2020 and early 2021 presented new and additional challenges: shorter, colder days, weariness of the restrictions, and increased financial pressures with high rates of unemployment and redundancy[34]. In addition, positive cases in childcare or school settings can result in children being required to isolate for 10 days without being allowed out. The importance of supporting families to maintain or increase activity for young children within the home and in accessible outdoor environments becomes more prominent. Families need support to establish healthy revised routines and manageable healthy rules for snacks and screen time during periods of restriction. This must be presented in a context of supporting parents and not adding guilt or burden to parents during a period of stress. A recent Ipsos Mori report, which included an online survey of 1,000 parents of 0-5s, found 70% of parents felt judged by others and almost half felt this negatively impacted on their mental health [35]. There is a role here for all sectors of health, social care and education including health visitors, children's centres, early years providers, GPs, social services, local authority public health teams and Public Health England. Further, there is a role for research with pre-schoolers and 2020 school starters to be expanded [36] to understand the impact on their physical and social development, and particularly their risk of obesity, as they start school.

Strengths and limitations

In-depth interviews enabled us to gain novel insights into the experiences of parents and pre-school children during lockdown and restrictions, though we acknowledge the limitation of investigating pre-

For peer review only - http://bmjopen.bmj.com/site/about/guidelines.xhtml

UOB Confidential & Sensitive

For peer review only

school children's experiences by asking their parents, rather than involving pre-school children directly. A strength of the study was the ethnic and socioeconomic diversity of the sample from two regions of England, although this was a relatively small (n=20) and educated sample. Parents volunteering for interview may have had more interest in the topic or had different experiences to those who did not volunteer. We acknowledge that the use of remote interviews could have compromised participants' ability to express themselves and non-verbal clues may have been missed[37]. Other primary caregivers were eligible for the study, but none were recruited. Rigour was achieved by detailed data analysis and analytical decisions being shared with all team members to achieve credibility.

Conclusions

While some positive changes were reported, there were wide-spread examples of lack of routines, habits and boundaries which, at least in the short-term, were likely to have been detrimental for child health and development. Guidance and support for parents and families on how to maintain healthy routines and compensate for future COVID-19 restrictions could be valuable to protect child health and ensure that unhealthy habits are not adopted.

For peer review only - http://bmjopen.bmj.com/site/about/guidelines.xhtml

UOB Confidential & Sensitive

For peer review only

Licence statement

I, the Submitting Author has the right to grant and does grant on behalf of all authors of the Work (as defined in the below author licence), an exclusive licence and/or a non-exclusive licence for contributions from authors who are: i) UK Crown employees; ii) where BMJ has agreed a CC-BY licence shall apply, and/or iii) in accordance with the terms applicable for US Federal Government officers or employees acting as part of their official duties; on a worldwide, perpetual, irrevocable, royalty-free basis to BMJ Publishing Group Ltd ("BMJ") its licensees and where the relevant Journal is co-owned by BMJ to the co-owners of the Journal, to publish the Work in BMJ Open and any other BMJ products and to exploit all rights, as set out in our licence.

Abbreviations

BAME	Black, Asian and Minority Ethnic
CO-SPYCE	Covid-19: Supporting Parents and Young Children during Epidemics (study)
COVID-19	Coronavirus Disease (also known as SARS-CoV-2)
GPs	General Practitioners
IMD	Index of Multiple Deprivation
UK	United Kingdom

Ethics approval and consent to participate

Ethical approval for the study was granted by University of Bristol Faculty of Health Sciences Research Ethics Committee. Participant consent was recorded on a separate audio file before the interview commenced.

For peer review only - http://bmjopen.bmj.com/site/about/guidelines.xhtml

UOB Confidential & Sensitive

Consent for publication

Consent to include anonymised information in publications was given by all participants.

Availability of data and materials

Anonymised study data will be made available via a University of Bristol repository.

Competing interests

The authors declare that they have no competing interests.

Funding

This work was funded by the NIHR School for Public Health Research (SPHR-PROG-CYP-WP3) and NIHR funding for the NAP SACC UK trial (2019-3426).

Authors' contributions

The study was conceived by JC, RK, SC, KW, HT, RB, KH, SS and RL. JC led the study with oversight from RL and RK. Interviews were conducted by JC, SC and KW. Coding of the data was performed by JC, HT and RB. JC, RK and RL produced the first draft of the manuscript, with all other authors providing critical review and intellectual content. All authors read and approved the final manuscript.

Acknowledgements

We are very grateful to all the parents who took part in the research interviews. The views expressed in this publication are those of the authors and not necessarily any of the funding bodies listed.

For peer review only

For peer review only - http://bmjopen.bmj.com/site/about/guidelines.xhtml

UOB Confidential & Sensitive

References

1 World Health Organization. Coronavirus disease (COVID-19) pandemic. 2020.https://www.who.int/emergencies/diseases/novel-coronavirus-2019 (accessed 7 Jul 2021).

2 Public Health England. Health matters: physical activity - prevention and management of long-term conditions. 2020. https://www.gov.uk/government/publications/health-matters-physical-activity/health-matters-physical-activity-prevention-and-management-of-long-term-conditions (accessed 7 Jul 2021).

3 Lawler R. New report shows scale of crisis in early years sector. Early Years Alliance. 2020. https://www.eyalliance.org.uk/news/2020/06/new-report-shows-scale-crisis-early-years-sector (accessed 7 Jul 2021).

4 Wu Z, McGoogan JM. Characteristics of and Important Lessons from the Coronavirus Disease 2019 (COVID-19) Outbreak in China: Summary of a Report of 72314 Cases from the Chinese Center for Disease Control and Prevention. *JAMA*. 2020;**323**:1239–42. doi:10.1001/jama.2020.2648

5 Foster C, Reilly J, Jago R, *et al.* UK Chief Medical Officers' Physical Activity Guidelines. 2019. https://assets.publishing.service.gov.uk/government/uploads/system/uploads/attachment_data/file/832868/uk-chief-medical-officers-physical-activity-guidelines.pdf (accessed 7 Jul 2021).

6 NHS UK. How much sleep do children need? 2017.https://www.nhs.uk/live-well/sleep-and-tiredness/how-much-sleep-do-kids-need/ (accessed 7 Jul 2021).

7 Hesketh KR, Griffin SJ, van Sluijs EMF. UK Preschool-aged children's physical activity levels in childcare and at home: A cross-sectional exploration. *International Journal of Behavioral Nutrition and Physical Activity* 2015;**12**:123. doi:10.1186/s12966-015-0286-1

8 Brazendale K, Beets MW, Weaver RG, *et al.* Understanding differences between summer vs. school obesogenic behaviors of children: The structured days hypothesis. *International Journal of Behavioral Nutrition and Physical Activity* 2017;**14**:1–14. doi:10.1186/s12966-017-0555-2

9 Clark T. 31% of shoppers have changed their primary supermarket in lockdown. https://www.retail-focus.co.uk/31-of-shoppers-have-changed-their-primary-supermarket-in-lockdown/. (accessed 7 Jul 2021).

For peer review only - http://bmjopen.bmj.com/site/about/guidelines.xhtml

UOB Confidential & Sensitive

10 Serdula MK, Ivery D, Coates RJ, *et al.* Do Obese Children Become Obese Adults? A Review of the Literature. *Preventive Medicine* 1993;**22**:167–77. doi:10.1006/pmed.1993.1014

11 NHS Digital. National Child Measurement Programme, England 2018/19 School Year. https://digital.nhs.uk/data-and-information/publications/statistical/national-child-measurement-programme/2018-19-school-year (accessed 7 Jul 2021).

12 Department for Education. Coronavirus (COVID-19) attendance in educational and early years settings in England – summary of returns to 17 April 2020. GOV.UK. 2020. https://assets.publishing.service.gov.uk/government/uploads/system/uploads/attachment_data/file/883173/COVID-19_attendance_in_education_settings_170420.pdf (accessed 7 Jul 2021).

13 Prime H, Wade M, Browne DT. Risk and resilience in family well-being during the COVID-19 pandemic. *American Psychologist* 2020;**75**:631–43. doi:10.1037/amp0000660

14 Royal College of Paediatrics and Child Health, Children & Young People's Engagement team. COVID-19 - research studies on children and young people's views. 2020. https://www.rcpch.ac.uk/resources/covid-19-research-studies-children-young-peoples-views (accessed 7 Jul 2021).

15 Dodd H, Westbrook J, Lawrence P. Co-SPYCE STUDY - 1ST UPDATE. https://emergingminds.org.uk/co-spyce-study-1st-update/. 2020. https://emergingminds.org.uk/co-spyce-study-1st-update/ (accessed 7 Jul 2021).

16 Public Health Scotland. COVID-19 Early years resilience and impact survey (CEYRIS). 2020. http://www.healthscotland.scot/publications/covid-19-early-years-resilience-and-impact-survey-ceyris (accessed 7 Jul 2021).

17 NAP SACC (UK): Nutrition and physical activity self-assessment for childcare. 2019. https://www.isrctn.com/ISRCTN33134697 (accessed 7 Jul 2021).

18 Guest G, MacQueen KM, Namey EE. *Applied Thematic Analysis*. Los Angeles, London, New Delhi, Singapore, Washington DC: Sage Publishing 2012.

For peer review only - http://bmjopen.bmj.com/site/about/guidelines.xhtml

UOB Confidential & Sensitive

19 Hesketh KR, Lakshman R, van Sluijs EMF. Barriers and facilitators to young children's physical activity and sedentary behaviour: a systematic review and synthesis of qualitative literature. *Obesity Reviews* 2017;**18**:987–1017. doi:10.1111/obr.12562

20 Carroll N, Sadowski A, Laila A, *et al.* The Impact of COVID-19 on Health Behavior, Stress, Financial and Food Security among Middle to High Income Canadian Families with Young Children. *Nutrients* 2020;**8**. doi:10.3390/nu12082352

21 Dunton GF, Do B, Wang SD. Early effects of the COVID-19 pandemic on physical activity and sedentary behavior in children living in the U.S. *BMC Public Health* 2020;**20**:1–13. doi:10.1186/s12889-020-09429-3

22 Moore SA, Faulkner G, Rhodes RE, *et al.* Impact of the COVID-19 virus outbreak on movement and play behaviours of Canadian children and youth: A national survey. *International Journal of Behavioral Nutrition and Physical Activity* 2020;**17**:1–11. doi:10.1186/s12966-020-00987-8

23 Moore SA, Faulkner G, Rhodes RE, *et al.* Impact of the COVID-19 virus outbreak on movement and play behaviours of Canadian children and youth: A national survey. *International Journal of Behavioral Nutrition and Physical Activity* 2020;**17**:85. doi:10.1186/s12966-020-00987-8

24 Zecevic CA, Tremblay L, Lovsin T, *et al.* Parental Influence on Young Children's Physical Activity. *International Journal of Pediatrics* 2010;**2010**:1–9. doi:10.1155/2010/468526

25 Idoiaga Mondragon N, Berasategi Sancho N, Dosil Santamaria M, *et al.* Struggling to breathe: a qualitative study of children's wellbeing during lockdown in Spain. *Psychology and Health* 2021;**36**:179–94. doi:10.1080/08870446.2020.1804570

26 Dellagiulia A, Lionetti F, Fasolo M, *et al.* Early impact of COVID-19 lockdown on children's sleep: A 4-week longitudinal study. *Journal of Clinical Sleep Medicine*. 2020;**16**:1639–40. doi:10.5664/jcsm.8648

27 Gardner B, Lally P, Wardle J. Making health habitual: The psychology of "habit-formation" and general practice. *British Journal of General Practice*. 2012;**62**:664–6. doi:10.3399/bjgp12X659466

28 Lally P, van Jaarsveld CHM, Potts HWW, *et al.* How are habits formed: Modelling habit formation in the real world. *European Journal of Social Psychology* 2010;**40**:998–1009. doi:10.1002/ejsp.674

For peer review only - http://bmjopen.bmj.com/site/about/guidelines.xhtml

UOB Confidential & Sensitive

For peer review only

29 Andrew A, Cattan S, Costa-Dias M, *et al.* Family time use and home learning during the COVID-19 lockdown. 2020. https://ifs.org.uk/publications/15038 (accessed 7 Jul 2021).

30 Rundle AG, Park Y, Herbstman JB, *et al.* COVID-19–Related School Closings and Risk of Weight Gain Among Children. *Obesity*. 2020;**28**:1008–9. doi:10.1002/oby.22813

31 Public Health England. National Child Measurement Programme 2020: Information for schools. 2020. https://assets.publishing.service.gov.uk/government/uploads/system/uploads/attachment_data/file/931777/NCMP_operational_guidance_ADDENDUM_2020.pdf (accessed 7 Jul 2021).

32 Sinha I, Bennett D, Taylor-Robinson DC. Children are being sidelined by covid-19. *BMJ* 2020;**369**. doi:10.1136/BMJ.M2061

33 Hefferon C, Taylor C, Bennett D, *et al.* Priorities for the child public health response to the COVID-19 pandemic recovery in England. *Archives of disease in childhood* 2021;**106**. doi:10.1136/ARCHDISCHILD-2020-320214

34 Thomas D. Unemployment rate hits highest level in three years. BBC News. 2020. https://www.bbc.co.uk/news/business-54520521 (accessed 7 Jul 2021).

35 Sweet O, Long J, Evans L, *et al.* State of the Nation: Understanding Public Attitudes to The Early Years. 2020. https://mk0royalfoundatcnhl0.kinstacdn.com/wp-content/uploads/2020/11/Ipsos-MORI-SON_report_FINAL_V2.4.pdf (accessed 7 Jul 2021).

36 Education Endowment Foundation. Impact of Covid-19 on primary school starters. 2020. https://educationendowmentfoundation.org.uk/projects-and-evaluation/projects/the-impact-of-covid-19-on-school-starters/ (accessed 7 Jul 2021).

37 Clarke V, Braun V. *Successful Qualitative Research: A Practical Guide for Beginners*. 1st ed. London: Sage 2013.

For peer review only

For peer review only - http://bmjopen.bmj.com/site/about/guidelines.xhtml

UOB Confidential & Sensitive

Table 1

Summary of topic guide used by interviewers to guide discussions during semi-structured interviews with parents of pre-school children. The full topic guide is available as supplementary material. This table was created by the co-authors for this publication.

Interview topics
Physical activity<br>– *Impact on activity in and out of house. More/less/same as before? Why?*
Sedentary activities<br>– *Impact on and types of activities. More/less/same as before? Why?*
Sleep behaviours<br>– *Impact on bedtimes, getting to sleep and staying asleep. More/less/same as before? Why?*
Eating<br>– *Impact on what and when food is eaten (mealtimes & snacks). More/less/ same as before? Why?*
Food purchasing<br>– *Amount, type, cost and access to food*
Food preparation<br>– *Changes to who, how, when food is prepared and why*

For peer review only

For peer review only - http://bmjopen.bmj.com/site/about/guidelines.xhtml
UOB Confidential & Sensitive

Table 2

Demographic characteristics of the parents of pre-school children who participated in a semi-structured interview (n=20). This table was created by the co-authors for this publication.

Characteristic Categories		N(%)
**Gender**	*Female*	16 (80)
	Male	4 (20)
**Age Group**	*21-25*	1 (5)
	26-30	7 (35)
	31-35	5 (25)
	36-40	2 (10)
	41-45	5 (25)
**Ethnicity**	*White*	12 (60)
	Asian or Asian British	4 (20)
	Black or Black British	1 (5)
	Mixed	3 (15)
**IMD Quintile** (1 = most deprived)	*1*	10 (50)
	2	2 (10)
	3	3 (15)
	4	3 (15)
	5	2 (10)
**Anyone in the household in paid employment**	*Yes*	17 (85)
	No	3 (15)
**Educated to degree level**	*Yes*	13 (65)
	No	7 (35)
**Single parent**	*Yes*	5 (25)
	No	15 (75)

For peer review only

UOB Confidential & Sensitive

For peer review only - http://bmjopen.bmj.com/site/about/guidelines.xhtml

Coronavirus Preschool Study: a qualitative study of the lived experience of final year pre-school children in the UK during the coronavirus (COVID-19) disruption – Interview Topic Guide

Introduction

- Hello [name], it's [name] from the University of Bristol/Birmingham/Glasgow speaking, how are you doing? Are you still ok to chat to me today? It will take up to an hour.
- Thank you so much for sparing your time to speak to me today.
- Before starting the interview, I'm going to talk a bit about the study. I'm then going to take your consent before we start the interview.
- Firstly, just to give a bit of background of the study, we want to find out about how pre-school children are doing during the coronavirus disruption, and what effects, if any, it is having on their mood and their physical activity and eating behaviours. Throughout the interview I will refer to coronavirus which some people call COVID-19.
- When answering the questions, please try to focus on your final year pre-school child rather than any other children in the household.
- We are interested in the whole period since mid-March, i.e. lockdown, easing of restrictions and now.
- Feel free to ask questions at any time.
- There are no wrong or right answers and you do not have to answer all of the questions if you do not want to.
- Anything that you tell me is confidential and will not be linked to you. The only reason for me needing to break confidentiality would be if you say something where I am concerned about harm to you or someone else.
- You can stop at any time and if you decide in the next two weeks that you do not want me to include what you said, just let me know.
- I have already sent you a copy of the consent form along with the participant information sheet. When I start the voice recorder, I'm just going to read out each statement on the consent form one by one and if you could please confirm that you're happy with each statement. This is something we have to do as part of the University ethics regulations.
- Do you have any questions before I start the recording? If it's ok, I will start recording now.

START RECORDING

- State interviewer name, participant name, participant ID number and date/time of interview.
- Read out each statement on the consent form. Ask participant to say 'Yes' after each statement if they are happy with the statement.
- Explain to the participant that you are now going to stop the audio-recording, and re-start recording for the main part of the interview.

STOP RECORDING

START RECORDING

State interviewer name, participant ID and date/time of interview (do NOT state participant name).

For peer review only - http://bmjopen.bmj.com/site/about/guidelines.xhtml

Background

Tell me about your household	• Name/age/gender of child focusing on for this interview<br>• Relationship of interviewee to child<br>• Age/gender of any other children<br>• Do you have a garden?
Is anyone in the house working ?	• Any keyworkers ?<br>• Anyone working from home; furloughed?
Has anyone in the household had coronavirus ?	• Tested or untested

Childcare

Remind participant that the focus of the interview is on the final year preschool child (not on other children in the family).

**Before** the coronavirus disruption, what were you doing for childcare?	• Setting (e.g. nursery, childminder, family members)<br>• How many days/hours per week of childcare?
**During** the lockdown when nurseries were shut except to key workers, did your child attend any childcare?	• If so, why? As much as before, or less?
How have you been managing childcare with the coronavirus disruption?	• Who is/has been looking after the children?<br>• If your nursery has **re-opened**, is your child currently attending any childcare? Is this as more or less than before?<br>• If your child has not returned to childcare, can you tell me why?<br>• If you child has returned to childcare, what changes have been made in childcare because of coronavirus? (e.g. attending part-time, parents not allowed in, smaller groups, changes to food provision)

Your child's mood and how they have been feeling

note to interviewer – we are interested in the whole period since mid-March, i.e. lockdown, easing of restrictions and now

How has your child been feeling about the coronavirus restrictions?	• What effects (if any) do you think the restrictions have had on your child's well-being?<br>• Have the restrictions had any effect on your child's mood and feelings? (positive or negative)<br>• Have you noticed any changes at all in your child's behaviour? (positive or negative)<br>• What is the impact on the child of any reduced social contact with other children, friends and family?

For peer review only - http://bmjopen.bmj.com/site/about/guidelines.xhtml

Physical activity

We're interesting in how the lockdown and the social distancing restrictions that are happening now might have affected young children's activities they do when they're moving around or sat still. By moving around, I mean anything from: Pottering (e.g. Lego, helping around the house, small-world play, dressing up); On the Go: (e.g. playing in the garden, dancing, hide and seek, playing on the furniture, rough and tumble); and Huff and Puff: (e.g. running games, trampolining, scooting, dancing).

note to interviewer – we are interested in the whole period since mid-March, i.e. lockdown, easing of restrictions and now

What activities has your child been doing during the coronavirus disruption?	• Has the coronavirus disruption had any effect on how active your child is?<br>• What has changed? What has stopped?
During the time since lock-down started in March has your child been going out of your home and garden (if they have one)?	• If not, why not?<br>• Has this changed as lockdown has been eased?
Do you think your child is currently more or less active than before the disruption?	• Why?<br>• How do you think any changes will affect how active your child is in the future?<br>• Will you re-start any activities that you can no longer do?<br>• Will you carry on doing any new activities?
What sitting activities has your child been doing during the coronavirus disruption? (e.g. screen time, educational activities, TV, games, social interaction)	• Has there been any change in the amount of time the child spends sitting?<br>• Have there been any changes in screen time rules or supervision of screen time in your house during the disruption?<br>• How do you think any changes will affect future screen use?
Do you think the changes since March due to coronavirus have affected your child's sleep?	• In what way?

School

How are you feeling about your child starting school in September?	• How is your child feeling?<br>• Any thoughts about whether child will start in September, or whether you may delay starting school?
Are there any things you would have been doing to help prepare the child for starting school that you aren't able to do because of the disruption?	• E.g. visits to new school, end of preschool special events<br>• What could help with the transition during this time of uncertainty?

Nutrition

note to interviewer – we are interested in the whole period since mid-March, i.e. lockdown, easing of restrictions and now

For peer review only - http://bmjopen.bmj.com/site/about/guidelines.xhtml

Have there been any changes in what your child has been eating because of the coronavirus disruption?	• E.g. changes in quantity of food, types of food?<br>• Any changes in how you get your food, what food you can get, how much you spend?<br>• Changes in snacking/treats?<br>• Any changes to food preparation at home? E.g. more/fewer take away meals, cooking with child, cooking from scratch?<br>• Changes in fruit and vegetable consumption?
Overall, do you think that your child is eating more or less healthily than before the disruption?	• Why?<br>• Thinking about the future, do you think that any changes to your child's eating will continue?

Demographic questions

- We would like to ask you some questions about you and your children. If you do not wish to answer, please just say so.
- If you don't mind telling me, what is your age?
- How would you describe your ethnicity?
- Out of the following options, what is your current employment status?
  - Student
  - Stay at home parent/caregiver
  - Full-time
  - Part-time
  - Unemployed
  - Furloughed
- What is your highest level of education? Options:
  - Up to GCSEs/GCEs/O levels or similar
  - A levels/NVQs/GNVQs
  - First degree/diploma/HNC/HND
  - Higher degree (e.g. MSc, PhD)
- Finally, how did you find out about this research study i.e. where did you see the study advert?

End

- That's all the questions I have for you today. Do you have any questions for me?
- I will stop recording the interview now.

STOP RECORDING

Thank the participant and explain that a voucher will be emailed to them within 7 days.

For peer review only

For peer review only - http://bmjopen.bmj.com/site/about/guidelines.xhtml

Standards for Reporting Qualitative Research (SRQR)*

http://www.equator-network.org/reporting-guidelines/srqr/

Title and abstract

	Page/line no(s).
**Title** - Concise description of the nature and topic of the study Identifying the study as qualitative or indicating the approach (e.g., ethnography, grounded theory) or data collection methods (e.g., interview, focus group) is recommended	1
**Abstract** - Summary of key elements of the study using the abstract format of the intended publication; typically includes background, purpose, methods, results, and conclusions	1-2

Introduction

**Problem formulation** - Description and significance of the problem/phenomenon studied; review of relevant theory and empirical work; problem statement	4-5
**Purpose or research question** - Purpose of the study and specific objectives or questions	5

Methods

**Qualitative approach and research paradigm** - Qualitative approach (e.g., ethnography, grounded theory, case study, phenomenology, narrative research) and guiding theory if appropriate; identifying the research paradigm (e.g., postpositivist, constructivist/ interpretivist) is also recommended; rationale**	6
**Researcher characteristics and reflexivity** - Researchers' characteristics that may influence the research, including personal attributes, qualifications/experience, relationship with participants, assumptions, and/or presuppositions; potential or actual interaction between researchers' characteristics and the research questions, approach, methods, results, and/or transferability	6
**Context** - Setting/site and salient contextual factors; rationale**	5
**Sampling strategy** - How and why research participants, documents, or events were selected; criteria for deciding when no further sampling was necessary (e.g., sampling saturation); rationale**	5-6
**Ethical issues pertaining to human subjects** - Documentation of approval by an appropriate ethics review board and participant consent, or explanation for lack thereof; other confidentiality and data security issues	7
**Data collection methods** - Types of data collected; details of data collection procedures including (as appropriate) start and stop dates of data collection and analysis, iterative process, triangulation of sources/methods, and modification of procedures in response to evolving study findings; rationale**	6

For peer review only - http://bmjopen.bmj.com/site/about/guidelines.xhtml

For peer review only

**Data collection instruments and technologies** - Description of instruments (e.g., interview guides, questionnaires) and devices (e.g., audio recorders) used for data collection; if/how the instrument(s) changed over the course of the study	6, 23
**Units of study** - Number and relevant characteristics of participants, documents, or events included in the study; level of participation (could be reported in results)	7, 24
**Data processing** - Methods for processing data prior to and during analysis, including transcription, data entry, data management and security, verification of data integrity, data coding, and anonymization/de-identification of excerpts	6
**Data analysis** - Process by which inferences, themes, etc., were identified and developed, including the researchers involved in data analysis; usually references a specific paradigm or approach; rationale**	6
**Techniques to enhance trustworthiness** - Techniques to enhance trustworthiness and credibility of data analysis (e.g., member checking, audit trail, triangulation); rationale**	6

Results/findings

**Synthesis and interpretation** - Main findings (e.g., interpretations, inferences, and themes); might include development of a theory or model, or integration with prior research or theory	7-12
**Links to empirical data** - Evidence (e.g., quotes, field notes, text excerpts, photographs) to substantiate analytic findings	7-12

Discussion

**Integration with prior work, implications, transferability, and contribution(s) to the field -** Short summary of main findings; explanation of how findings and conclusions connect to, support, elaborate on, or challenge conclusions of earlier scholarship; discussion of scope of application/generalizability; identification of unique contribution(s) to scholarship in a discipline or field	13-15
**Limitations** - Trustworthiness and limitations of findings	15-16

Other

**Conflicts of interest** - Potential sources of influence or perceived influence on study conduct and conclusions; how these were managed	18
**Funding** - Sources of funding and other support; role of funders in data collection, interpretation, and reporting	18

*The authors created the SRQR by searching the literature to identify guidelines, reporting standards, and critical appraisal criteria for qualitative research; reviewing the reference lists of retrieved sources; and contacting experts to gain feedback. The SRQR aims to improve the transparency of all aspects of qualitative research by providing clear standards for reporting qualitative research.

For peer review only - http://bmjopen.bmj.com/site/about/guidelines.xhtml

**The rationale should briefly discuss the justification for choosing that theory, approach, method, or technique rather than other options available, the assumptions and limitations implicit in those choices, and how those choices influence study conclusions and transferability. As appropriate, the rationale for several items might be discussed together.

Reference:

O'Brien BC, Harris IB, Beckman TJ, Reed DA, Cook DA. **Standards for reporting qualitative research: a synthesis of recommendations.** *Academic Medicine*, Vol. 89, No. 9 / Sept 2014 DOI: 10.1097/ACM.0000000000000388

For peer review only
